# Fire Smoke Elevated the Carbonaceous PM_2.5_ Concentration and Mortality Burden in the Contiguous U.S. and Southern Canada

**DOI:** 10.1021/acs.est.5c01641

**Published:** 2025-06-12

**Authors:** Zhihao Jin, Gonzalo A. Ferrada, Danlu Zhang, Noah Scovronick, Joshua S. Fu, Kai Chen, Yang Liu

**Affiliations:** † Gangarosa Department of Environmental Health, Rollins School of Public Health, 1371 Emory University, Atlanta, Georgia 30322, United States; ‡ Deparent of Civil and Environmental Engineering, 4292 University of Tennessee, Knoxville, Tennessee 37996, United States; § Deparent of Biostatistics, Rollins School of Public Health, Emory University, Atlanta, Georgia 30322, United States; ∥ Global Systems Laboratory, NOAA Earth System Research Laboratories, Boulder, Colorado 80305, United States; ⊥ Department of Environmental Health Sciences, 50296 Yale School of Public Health, New Haven, Connecticut 06510, United States; # Yale Center on Climate Change and Health, Yale School of Public Health, New Haven, Connecticut 06510, United States

**Keywords:** wildland fire, mortality, fine particulate matter, prescribed fire, PM_2.5_ speciation, United States, Canada

## Abstract

Despite emerging evidence on the health impacts of fine particulate matter (PM_2.5_) from wildland fire smoke, the specific effects of PM_2.5_ composition on health outcomes remain uncertain. We developed a three-level, chemical transport model-based framework to estimate daily full-coverage concentrations of smoke-derived carbonaceous PM_2.5_, specifically organic carbon (OC) and elemental carbon (EC), at a 1 × 1 km^2^ spatial resolution from 2002 to 2019 across the contiguous U.S. (CONUS) and Southern Canada (SC). A 10-fold random cross-validation confirmed robust performance, with daily *R* ^2^ = 0.77 (OC) and 0.80 (EC) in the smoke-off scenario and 0.67 (OC) and 0.71 (EC) in the smoke-on scenario, and exceeded 0.90 at the monthly scale after residual adjustment. Modeling results indicated that increases in wildland fire smoke have offset approximately one-third of the improvements in background air quality. In recent years, wildland fire smoke has become more frequent and carbonaceous PM_2.5_ concentrations have intensified, especially in the Western CONUS and Southwestern Canada. Wildfire season is also starting earlier and lengthens throughout the year, leading to more population being exposed. We estimated that long-term exposure to fire smoke carbonaceous PM_2.5_ is responsible for approximately 7455 and 259 non-accidental deaths annually in the CONUS and SC, respectively, with associated annual monetized damage of 68.3 billion USD for the CONUS and 1.9 billion CAD for SC. The Southeastern CONUS, where prescribed fires are prevalent, contributed most to these health impacts and monetized damages. Our findings offer critical insights to inform policy development and assess future health burdens associated with fire smoke exposure.

## Graphical abstract

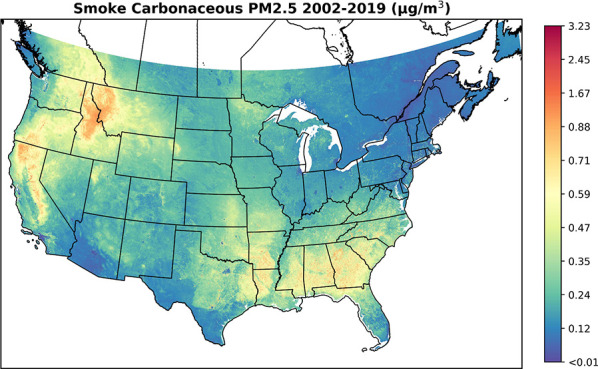

## Introduction

Over the past half-century, wildland fire activity has significantly increased in not only the U.S. but also other temperate and high-latitude fire-prone ecosystems, including those in Canada and Europe.^1,2^ Notably, human-induced climate change was responsible for an additional 4.2 million hectares of forest fire area between 1984 and 2015, compared to the area expected to be burned under natural climate variability alone.^3^ As a result, large-scale wildland fire events have become more frequent and intense, and fire seasons have lengthened in the contiguous U.S. (CONUS) in recent decades. Previous research has indicated that wildland fire smoke has contributed to nearly 25% of the ambient fine particulate matter (PM_2.5_, particles with a diameter of less than 2.5 μm) across the U.S. in recent years, and up to 50% in certain Western U.S. regions.^4^

One of the primary wildfire management strategies is prescribed burning. Prescribed fires not only reduce the biomass available for subsequent wildfires, but they also support carbon sequestration, facilitate ecological resilience, and play a critical role in restoring fire-adapted ecosystems that have been degraded due to decades of fire exclusion.^5,6^ Over 65% of the prescribed burn areas are in the Southeastern U.S.^7^ This frequent application of prescribed fires has resulted in elevated PM levels in the region.^8^ In the context of climate change, the growing reliance on prescribed burning to control wildfires has increased smoke emission from these burns, which have emerged as a significant public health concern, particularly in the Southeastern U.S.^9,10^ The National Prescribed Fire Acts (116th and 118th Congress) emphasize the importance of public health and safety risks associated with the expanded use of prescribed fires. However, it states that smoke from prescribed fires is generally less harmful and of shorter duration compared to wildfire smoke, stating that it exposes children to fewer adverse health effects.^11^ Such a statement, however, is based on limited research, which may lead to an underestimation of prescribed burning’s health risks.

As global warming continues to escalate wildfire activity, the negative impacts of smoke on air quality and public health are likely to worsen in the future.^12^ Fire smoke contains considerable amount of PM_2.5_, significantly deteriorating the air quality in downwind communities that are tens to hundreds of kilometers away.^13^ Smoke PM_2.5_ is characterized by substantial concentrations of carbonaceous matter, including organic carbon (OC) and elemental carbon (EC), which are produced by the combustion and incomplete burning of organic materials such as wood, leaves, and other vegetation. This distinguishes fire smoke PM_2.5_ from typical ambient PM_2.5_, which tends to present greater oxidative potential.^14–16^ As a result, smoke PM_2.5_ with unique chemical profile may alter the overall composition and toxicity of ambient PM_2.5_ in regions impacted by fire smoke.

While numerous studies have linked exposure to PM_2.5_ with various adverse health impacts, epidemiological research linking exposure to fire smoke PM_2.5_ with adverse health outcomes is still in its early stage.^17–20^ Long-term exposure to smoke PM_2.5_ has been linked to all-cause mortality in the CONUS, particularly among vulnerable populations such as the elderly.^21^ It is estimated that 11 415 non-accidental deaths per year in the CONUS can be attributed to smoke PM_2.5_, with cardiovascular diseases contributing the most.^21^ Short-term exposure to wildfire smoke PM_2.5_ has been associated with increased risks of respiratory morbidity, mental health issues, and excess mortality.^22–25^ However, evidence on the health effects of different chemical components of smoke PM_2.5_ remains sparse. For example, OC has been identified to be an important component influencing PM_2.5_ toxicity to several reactions harming organic systems and a key contributor to all-cause mortality.^26–28^ EC, due to its small size, can penetrate deeply into the respiratory tract and serve as a transporter for various toxic substances.^29^ Because OC and EC are the main products of biomass burning, quantifying their concentrations is critical for elucidating the toxicity profile of smoke PM_2.5_ and informing health studies.

Research on the health effects of smoke PM_2.5_ has been hindered due to the scarcity of long-term exposure data, especially data with comprehensive spatial coverage and high spatial-temporal resolution. Most epidemiological studies on smoke PM have relied on local ground-based monitoring stations, satellite images, uncalibrated chemical transport model (CTM) simulations or simple classifications of smoke-affected areas to investigate the health impacts of fire smoke.^30–35^ These methods were either did not quantify smoke-specific PM or based on coarse resolution smoke estimates, potentially introducing exposure misclassification. For instance, Kiser et al. classified smoke days via recorded events from a local air quality management division to investigate whether wildfire smoke modified the association between PM exposure with asthma visits. A key limitation of the study was the inability to isolate smoke-specific PM from other sources, potentially underestimating smoke-related health impacts.^34^ Liu et al. employed simulations of daily wildfire smoke PM_2.5_ from the GOES-Chem CTM at a solution of 0.5° × 0.65° to investigate risk of hospital admissions.^35^ Although CTMs can incorporate emissions, atmospheric chemistry, and meteorology to provide full spatial and temporal coverage and allow for separation of smoke PM_2.5_, the simulations has uncertainties from the input emission inventories and lacked sufficient spatial resolution for assess localized smoke exposure.^36^ Such limitations underscore the need for more refined CTM-based models that estimate smoke-specific PM_2.5_ at high spatiotemporal resolution.

Emerging research has shown great promise to generate long-term and high-resolution smoke PM_2.5_ concentrations by calibrating CTM simulations. For instance, Cleland et al. tested the model performance of predicting 1 × 1 km^2^ wildfire smoke PM_2.5_ based on CTMs simulations and different combinations of concentration data sets.^37^ The model that fused ground-based observations, satellite aerosol optical depth (AOD)-derived concentrations and CTMs simulations provided the best estimate (*R* ^2^ = 0.71) in fire-impacted regions, highlighting the importance of integrating multiple data sets. Similarly, Zhang et al. developed CMAQ-based models to estimate daily 1 × 1 km^2^ smoke PM_2.5_ total mass, which achieved strong model performance with *R* ^2^ of 0.75 and 0.68 in smoke-impacted regions and non-smoke regions, respectively.^38^ Nevertheless, few studies have adopted CTM-based models to estimate smoke PM_2.5_ speciation with high spatial and temporal resolution. This is largely because CTM simulations for PM_2.5_ speciation often face higher uncertainties compared to those for total PM_2.5_ mass, demanding more advanced calibration techniques.^39,40^ To address this challenge, a multi-step modeling approach that progressively refines CTM-based predictions by integrating ground-based measurements, satellite retrievals, and auxiliary data sets is needed. One promising example is the superlearner approach, a widely used approach that systematically combines outputs from multiple base learners and often outperforms any single model.^41–43^ Such a stepwise strategy is crucial for accurately capturing smoke PM_2.5_ speciation, as each stage refines the calibration and yields more reliable exposure estimates.

In this study, we developed a three-level, CTM-based machine learning model framework to estimate daily concentrations of fire smoke carbonaceous PM_2.5_, specifically OC and EC, at 1 × 1 km^2^ spatial resolution from 2002 to 2019 with full coverage across the CONUS and Southern Canada (SC). The three levels consist of (1) base models producing initial predictions, (2) a super learner integrating those outputs, and (3) a spatiotemporal residual adjustment based on the generalized additive model. This framework integrated information from CMAQ simulations of PM_2.5_ mass and speciation, ground-based observations and multiple auxiliary spatial and spatiotemporal data sets. This innovative approach allows us to fill important research gaps described above, namely, to differentiate exposure by specific carbonaceous constituents of smoke PM_2.5_, and to estimate fire smoke-related health burdens over the long term. By leveraging the high spatial and temporal resolution of our model predictions, we analyzed the spatiotemporal patterns in both the frequency of smoke impact and the concentrations of carbonaceous PM_2.5_ from fire smoke. Given the regional difference in fire regimes, meteorological conditions, and baseline air quality, we also examined how smoke carbonaceous PM_2.5_ impacts vary by climate region. Furthermore, we estimated the populations exposed to fire smoke. Lastly, we investigated the impacts of long-term exposure to fire smoke on mortality burden and associated monetized damages.

## Materials and Methods

### Study Domain and Period

Our study domain included the CONUS and SC (Figure 1). The daily predictions were developed at 1 × 1 km^2^ spatial resolution. In total, our modeling grid included 9 115 328 1 × 1 km^2^ grid cells for the CONUS and 2 620 640 1 × 1 km^2^ grid cells for SC, from 2002 to 2019. The study domain was constraint by the EPA’s standard Community Multiscale Air Quality (CMAQ) 12US1 modeling domain, which covers the entire CONUS and extends into the SC included in our study. The study covers the entire population of the CONUS and the majority of the population in Canada (Figure S1). The CONUS exhibits highly heterogeneous population densities, with densely populated metropolitan areas located in the Northeast, Southeast, and parts of the West, and more sparsely populated regions in the central and western areas. In contrast, SC is characterized by a mix of urban centers (e.g., Toronto, Montreal, and Vancouver) and lower-density rural regions.

**Figure 1:**
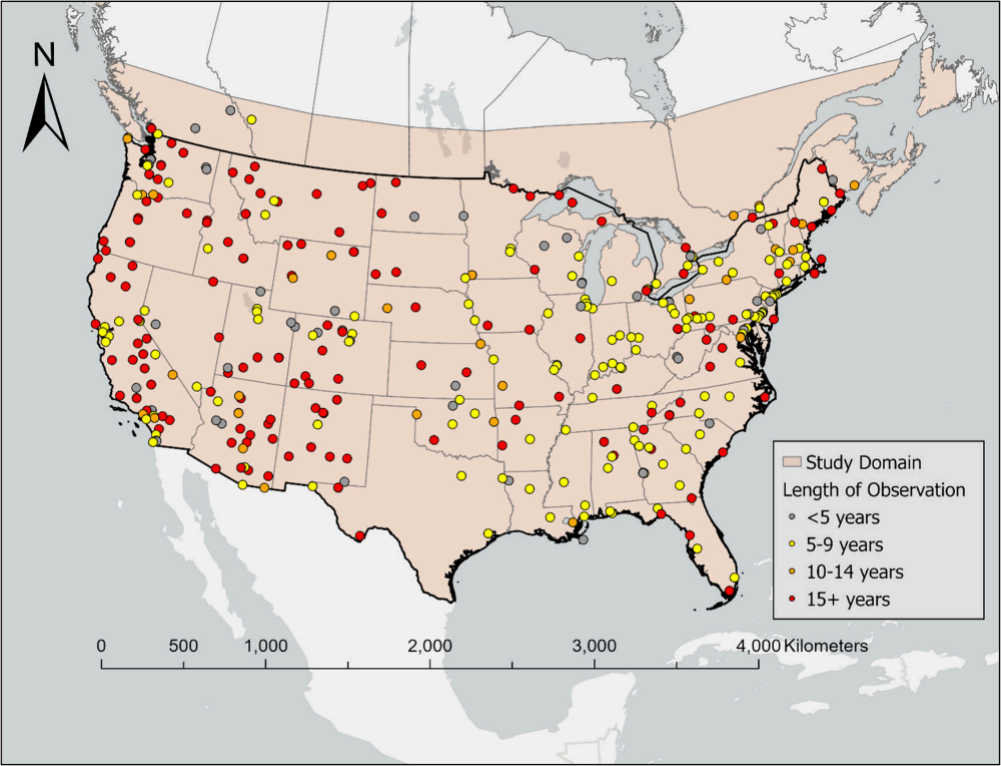
Study domain and ground-based monitoring networks of PM_2.5_ OC and EC.

### Ground-Based Carbonaceous PM_2.5_ Measurements

The ground-based measurements of carbonaceous PM_2.5_ (i.e., OC and EC) in the CONUS were obtained from a variety of sources, including the chemical speciation network (CSN) from Environmental Protection Agency (EPA), the National Park Service interagency monitoring of protected visual environments (IMPROVE) network, and the Southeastern Aerosol Research and Characterization Study (SEARCH) network.^44,45^ For SC, ground-based observations were provided by the National Air Pollution Surveillance (NAPS) program.^46^ In total, 401 monitors operated during our study period, with most stations operating for more than 5 years (Figure 1). Extremely high observations (>99.98th percentile) of PM_2.5_ OC and EC were excluded from the model training to minimize the outliers (99.98th percentile of OC and EC: 34.75 μg/m^3^ and 7.40 μg/m^3^). PM_2.5_ OC and EC stations are often colocated and collect samples synchronously every 3 days, with similar number of stations and observations overtime (Table S1). Our final data includes approximately 448 000 daily measurements for OC or EC. Detailed summary statistics of ground-based measurements for training data set are provided in Tables S2 and S3.

### Chemical Transport Model

The state-of-the-art CMAQ (www.epa.gov/cmaq) modeling system estimates atmospheric concentrations of numerous chemicals and aerosols, including ozone (and its precursors), PM_2.5_, and deposition of harmful chemical species.^47^ CMAQ is developed and maintained by the U.S. Environmental Protection Agency (EPA), and it has been widely used for assessing air pollution, including evaluating policies and estimating impacts on human health.^48–50^ In this study, we used CMAQ version 5.3.2 and the data sets from the EPA’s air QUAlity TimE Series (EQUATES; www.epa.gov/cmaq/equates) project.^51^ EQUATES is a multiyear air quality modeling platform designed by U.S. EPA to support long-term trend analysis and regulatory assessments. It provides both the input data and simulation outputs necessary for running the CMAQ model over long time periods. Specifically, the inputs include meteorological fields such as temperature, pressure, humidity, and wind speed derived from simulations using the Weather Research and Forecasting (WRF) model version 4.1.1, and emissions from multiple sectors (e.g., vehicles, fires, oil and gas industries, residential wood combustion, aircraft, and shipping), which were developed using consistent methods across years.

In this study, we leveraged two sets of daily CMAQ simulations at 12 × 12 km^2^ spatial resolution for the period of 2002–2019: (1) the baseline CMAQ simulation results from EQUATES, which includes all emission sources, and (2) a sensitivity simulation conducted using the same configurations (Table S4) and inputs, except by not including fire emissions. Hereafter, these two sets of simulations are referred to as “fire-inclusive” and “fire-exclusive”, respectively. Wildland fires and prescribed fires in the “fire-inclusive” simulation were identified by integrating fire emissions inventories from the NOAA Hazard Mapping System (HMS), the Incident Status Summary database, the Monitoring Trends in Burn Severity database, and the Geospatial Multi-Agency Coordination system.^52–54^ In addition, the simulation include cropland fires and grassland fires. Emissions from four fire types are combined to quantify the aggregated impact of fire smoke.

### Classification of Smoke-Impacted Areas

To isolate the specific contribution of fire smoke to PM_2.5_ OC and EC concentrations, we classified the grid cells in the study domain into two daily smoke scenarios: a background scenario without fire smoke impact (“smoke-off”) and a fire smoke-impacted scenario (“smoke-on”). All grid days were matched with the fire-exclusive CMAQ simulations to establish baseline conditions without smoke influence. Those grid days classified as smoke-on were also matched spatially and temporally with fire-inclusive CMAQ simulations. This allowed separate modeling and prediction for both scenarios to quantify smoke contributions at each smoke-on grid day by subtracting smoke-off concentrations from smoke-on concentrations.

We first calculated the smoke contribution to PM_2.5_ mass by subtracting fire-exclusive CMAQ simulations from the fire-inclusive CMAQ simulations. The ratio of smoke PM_2.5_ mass was then determined by dividing smoke contribution by the fire-inclusive PM_2.5_ mass from CMAQ simulations, as follows:

1 $${{\mathrm{PM}}_{2.5}}_{\mathrm{smoke}}^{\mathrm{CMAQ}}={{\mathrm{PM}}_{2.5}}_{\mathrm{fire‐inclusive}}^{\mathrm{CMAQ}}-{{\mathrm{PM}}_{2.5}}_{\mathrm{fire‐exclusive}}^{\mathrm{CMAQ}}$$

2 $$\mathrm{smoke}{\mathrm{PM}}_{2.5}\mathrm{ratio}={{\mathrm{PM}}_{2.5}}_{\mathrm{smoke}}^{\mathrm{CMAQ}}/{{\mathrm{PM}}_{2.5}}_{\mathrm{fire‐inclusive}}^{\mathrm{CMAQ}}$$

The smoke scenarios were defined based on the smoke PM_2.5_ mass ratio and the NOAA HMS smoke plume maps.^52^ These maps are generated by trained satellite analysts who manually integrate data from multiple satellite sensors to identify and outline areas affected by smoke, which depict daily spatial extent of smoke plumes originating from fires across North America. On a given day, grid cells that either fell within the HMS smoke plume polygon or had a smoke ratio greater than a predefined threshold were considered to be impacted by fire smoke.

To determine this threshold, we conducted a sensitivity analysis using smoke ratios ranging from 1 to 20%. A 3% threshold was ultimately selected to define smoke-impacted areas because it successfully covered most observed fire spots across seasons during the study period (Figure S2). These fire spots were obtained from the NOAA HMS which detects daily locations of potential biomass burning locations (e.g., wildfires, prescribed burns, and agricultural fires) using satellite imagery from multiple sensors.^52^ Additionally, the classification yielded acceptable correlations between CMAQ simulations and ground-based observations (e.g., Correlation: 0.32–0.55 for smoke-on grid days; Table S5), and maintained sufficient sample sizes for training models under the smoke-on scenario.

Several factors help explain the moderate correlations between CMAQ-simulated and monitored OC/EC. First, CMAQ outputs represent 12 × 12 km^2^ grid cell-averages, so local peaks and rural baselines are averaged in the CMAQ. On the other hand, monitors sample at a single microenvironment, which is often located in populated areas. Second, monitoring networks obtain OC and EC by collecting a 24 h aerosol sample on quartz filters and analyzing it thermally and optically. The filter is weighed then progressively heated to drive off organic compounds and then heated in an oxidizing atmosphere to combust the remaining EC. Carbon released in each stage is measured and reported as carbon mass. CMAQ models EC as primary particles emitted from combustion sources, whereas OC comprises those primary emissions plus secondary organic aerosol produced through the model-simulated oxidation of volatile precursors.^47^ Third, the CMAQ inherits uncertainties from its input emission inventories, while filter-based measurements introduce their own uncertainties from sampling and analysis methods employed, shipping, and blank/artifact estimation and correction.^44^ These discrepancies underscore the importance of integrating CMAQ simulations with auxiliary predictors to achieve robust estimates.

### Auxiliary Predictors

To enhance model performance and predictive accuracy, we incorporated a wide range of auxiliary predictors in model development. These predictors included satellite-retrieved AOD, which measures the optical concentration of airborne fine particles. Cloud coverage was included because of its impact on AOD retrieval quality. Smoke plume information, including duration and density, characterized daily fire smoke impact. We also incorporated meteorological variables (e.g., temperature, humidity, and wind speed) to account for their role in pollutant transport and dispersion. Vegetation indices such as NDVI and EVI, biogenic emissions, and land cover types were used to represent fuel availability and biogenic emission sources. Additional predictors such as population density, road density, elevation, and human footprint index served as proxies for human activity, traffic emissions, and topological characteristics. Finally, spatial coordinate and time trend terms were added to capture variation not explained by other predictors. These variables have been found to be important predictors in prior studies.^38,55–57^

To ensure spatial consistency, all predictors at different spatial resolutions were rescaled and aligned into the 1 × 1 km^2^ grid cells obtained from the Multi-Angle Implementation of Atmospheric Correction (MAIAC) data set, which served as the grid template for the MAIAC aerosol optical depth (AOD) measurements.^58^ Daily ground-based measurements of PM_2.5_ OC and EC were assigned to their collocated grid cells. Detailed descriptions of the data sources and process steps are provided in section S1 of the Supporting Information.

### Modeling Framework

After aggregating the raw measurements for OC and EC, we applied the Synthetic Minority Oversampling Technique (SMOTE) to oversample the underrepresented high-concentration measurements, improving model learning and performance in capturing extreme smoke pollution events.^59^ The enriched training data sets were then classified into smoke-off and smoke-on scenarios. To clarify, samples classified as smoke-on were excluded from training the smoke-off models. Conversely, smoke-off models produced predictions for every grid day, whereas smoke-on models were only used for smoke-on grid days. For both PM_2.5_ OC and EC, and both smoke scenarios, we employed the proposed Residual Adjusted Super Learner (RASL) (Figure 2), which integrates the strengths of multiple modeling approaches through a three-level modeling framework. At level 1, four base machine learning models were trained using cross-validated predictions and generated daily predictions. These predictions were then incorporated using a meta-learner algorithm at level 2 to generate fused predictions.^42^ This ensemble approach takes advantage of the predictive ability across base learners, which typically outperforms any single model. Finally, at level 3, generalized additive models (GAMs) were trained at the monthly level to adjust the remaining spatiotemporal residuals of the monthly averaged concentrations. This GAM step enabled us to correct for smooth, spatially varying biases that might be overlooked in level 1 and 2, thereby enhancing the robustness and accuracy of our long-term estimates across the entire 1 × 1 km^2^ grid from 2002 to 2019. In summary, RASL not only leverages the ensemble strength of the super learner but also addresses the spatiotemporal residuals, offering a robust approach to model smoke carbonaceous PM_2.5_. Details of the modeling framework are provided in section S2 of the Supporting Information.

**Figure 2:**
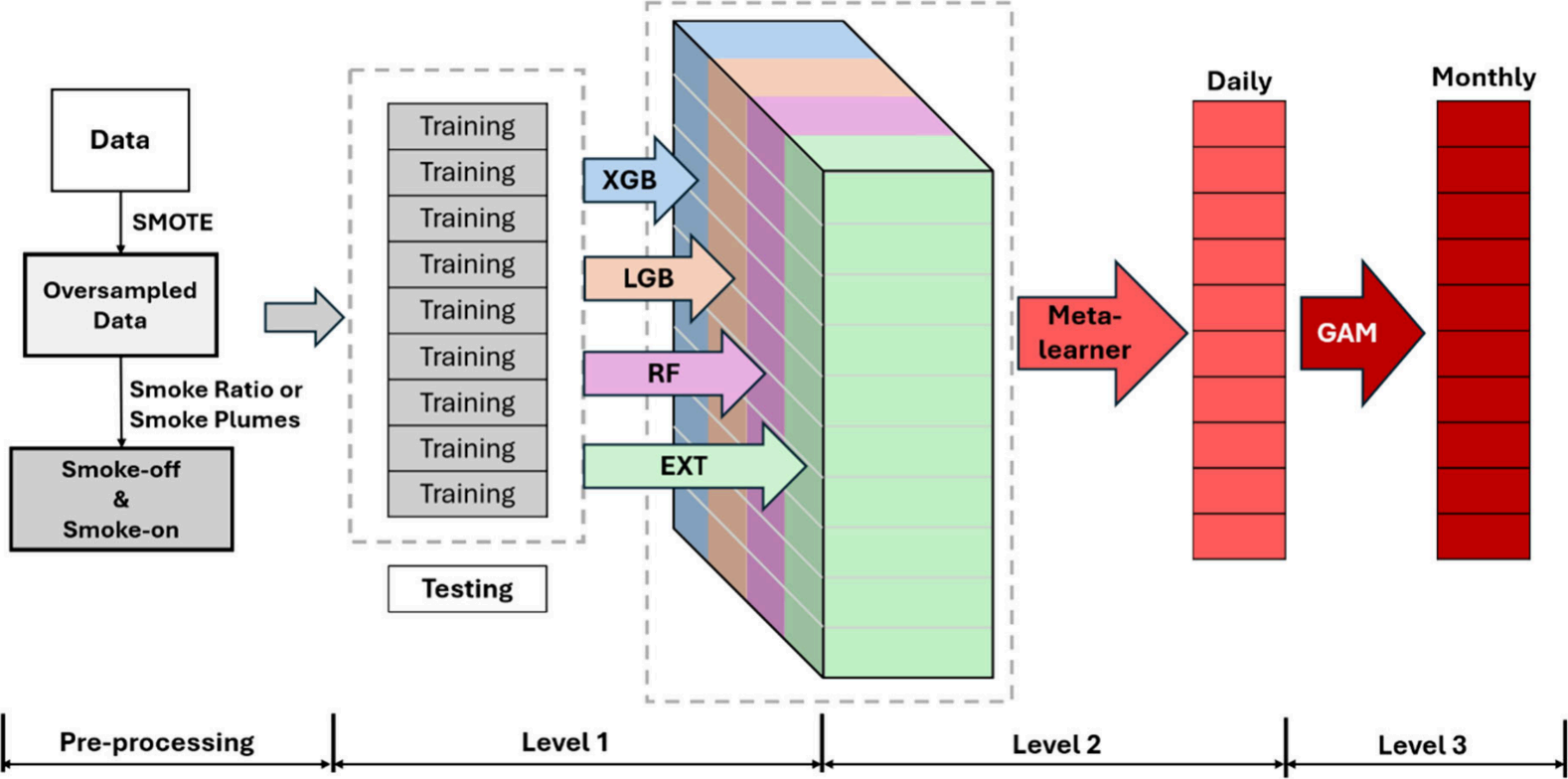
Modeling framework of the three-level residual adjusted super learner (RASL).

### Model Performance Evaluation

We conducted a three-stage cross-validation (CV) to evaluate the model performance at each level of the RASL framework. Three types of CV were employed: 10-fold random CV, 10-fold clustered spatial CV, and leave-one-year-out temporal CV. The clustered spatial CV better tests the model’s predictive ability when a large group of monitoring networks is missing instead of a single monitor.^60,61^ Prediction accuracy was evaluated using three metrics: the coefficient of determination (*R* ^2^), root-mean-square error (RMSE), and slope. To further evaluate the model at different time scales, we averaged daily estimations into monthly and annual values, allowing us to capture both long-term trends and short-term fluctuations, important for long-term cohort studies and short-term analyses. Details of the CV experiments are provided in section S3 of the Supporting Information. Ranked feature importance is also reported using impurity-based (for random forest and extremely randomized trees) and gain-based (for extreme gradient boosting and light gradient boosting machine) methods.

### Calculation of Mortality Burden and Monetized Damage

We employed different methods in the CONUS and SC to calculate the non-accidental mortality and monetized damages attributable to fire smoke carbonaceous PM_2.5_. For the CONUS, Ma et al. provided the monthly non-accidental mortality rates across different concentration bins of 12-month averaged smoke PM_2.5_ in the CONUS.^21^ The bins were defined based on percentile ranges of the concentrations. The monthly mortality rates were then multiplied by 12 months to estimate the annual mortality rate (Table S6). The annual mortality rate, along with each year’s smoke concentrations and population data, was used to estimate the following year’s deaths. To reflect uncertainty in estimating attributable mortality, we employed a Markov chain Monte Carlo (MCMC) that varies the mortality rates while holding exposure and population constant. Specifically, for each concentration bin we generated 10 000 random draws according to the published point estimate and 95% confidence interval (CI). For each calendar year we report the ensemble median and the 2.5th–97.5th percentiles as the point estimate and 95% CI, respectively. Because Ma et al.’s study is specific to smoke PM_2.5_ mass instead of speciation, we also performed two additional sensitivity analyses in CONUS to demonstrate the range of plausible mortality burdens. In the first sensitivity analysis, we rescaled the smoke-derived carbonaceous PM_2.5_ to represent different percentages (i.e., 50, 60, 70, 80, 90, and 95%) of the total smoke PM_2.5_ mass and repeated the MCMC calculation with median values extracted. The second analysis used PM_2.5_ composition-based hazard ratios for long-term exposure to OC and EC reported by Hao et al’s.^62^ The details of the second analysis are provided in section S4 of the Supporting Information.

To assess the monetized damages associated with these mortality estimates, we employed the Value of Statistical Life (VSL), as provided by the U.S. Department of Health and Human Services. VSLs reflect the monetary value that individuals are willing to pay to reduce the risk of death, thereby providing an economic perspective on mortality burden. We based our estimates on the 2013 VSL value and then adjusted it annually from 2003 to 2020 in accordance with HHS guidelines (Table S16), to account for inflation and changes in real income for the specific dollar year.^63^ The year-specific VSL values were multiplied by the estimated mortality attributable to fire smoke carbonaceous PM_2.5_ exposure in that year to provide an estimate of annual monetized damages and 95% CI.

For SC, we applied the Air Quality Benefits Assessment Tool (AQBAT) developed by Health Canada, which is designed to estimate the human health impacts and economic valuation of changes in Canada’s ambient air quality.^64^ AQBAT is a Microsoft Excel-based tool that allows users to define, run, review, and save inputs and outputs for specific air quality scenarios. It operates using data from 293 Census Divisions (CDs), based on the 2011 Canadian Census geography defined by Statistics Canada. AQBAT quantifies attributable morbidity or mortality from changes in air pollution concentration based on its internal population data, incidence rates, and concentration–response functions, which are derived from individual study or meta-analyses. For our study, we input the modeled annual average concentration of smoke carbonaceous PM_2.5_ as the change in PM_2.5_ concentration for each CD. AQBAT then applied the CRF for all-cause mortality due to chronic exposure from Crouse et al. to estimate attributable deaths, which found HRs of 1.10 (95% CI: 1.05, 1.15) for each 10 μg/m^3^ increase in concentrations of PM_2.5_.^65^ We then extracted the mean attributable deaths along with AQBAT’s built-in 2.5th and 97.5th percentiles estimates as the 95% CI. Additionally, AQBAT estimates the economic value of health impacts using the Canadian VSL provided by the Canada Policy Research Initiative (Table S17).^66^ Difference between the Canadian and U.S. VSL estimates is due to many factors, including currency year, purchasing power parity, and relative weighting of individual primary valuation studies. Annual monetized damages were calculated by multiplying the estimated attributable mortality by the corresponding VSL values.

## Results

### Model Performance

The CV results revealed strong model performance (Tables S7–S12). For daily level predictions, smoke-off models showed higher accuracy, with random CV *R* ^2^ values above 0.75 for base learners and 0.77 for meta-learners. Meta-learners for EC achieved average random CV *R* ^2^ values of 0.80 in smoke-off and 0.71 in smoke-on scenarios, while OC performance dropped in smoke-on with a reduced *R* ^2^ of 0.67 and increased RMSE of 1.20 μg/m^3^. Spatial CV showed low prediction errors for OC and EC in smoke-off (Figure S3), with RMSEs below 0.8 and 0.2 μg/m^3^, respectively. In contrast, smoke-on scenarios exhibited relatively higher RMSEs, especially in areas prone to fire smoke. For temporal CV, *R* ^2^ values were consistently higher for smoke-off scenarios (Figure S4), ranging between 0.65 and 0.85. In smoke-on scenarios, OC and EC displayed fluctuating *R* ^2^ values between 0.58 and 0.65, with notable declines in 2002. At monthly and annual levels, model performance improved significantly (Tables S7–S12). Adjustments to residuals through GAMs further enhanced accuracy (Table S13), with random CV *R* ^2^ values surpassing 0.91 for monthly and 0.97 for annual predictions. Most overestimations and underestimations observed at the daily level were reduced by averaging at the monthly level (Figure S5). Overall, the RASL model demonstrates reliable daily predictions across various CV experiments and further improvements in adjusting the long-term prediction residuals. Feature importance analysis (Figure S6) indicated CMAQ simulations of carbonaceous PM_2.5_ were primary predictors, with MAIAC AOD, urbanization factors, smoke plume density and duration, meteorological factors, and spatial and temporal characteristics also influential. Some land use types (i.e., shrubland and grassland) show higher importance in XGBoost models, which potentially play important roles in certain regions such as Texas where shrubland is the major vegetation type. These results illustrated the necessity of the meta-learner, which integrates the complementary strengths of the individual base learners in capturing influential predictors.

### Spatial and Temporal Patterns of Smoke Carbonaceous PM_2.5_

Figure S7 illustrates the average number of smoke days per year across our study domain. The central-south (i.e., Missouri, Arkansas, and Oklahoma) and southeastern (i.e., Alabama, Georgia, and Florida) regions of the CONUS exhibited the highest average number of smoke days (200+ days per year). The Western CONUS (i.e., California, Oregon, Idaho, and Montana), North-Central CONUS (i.e., North Dakota, South Dakota, and Minnesota) and adjacent areas in SC such as British Columbia also experienced a significant number of smoke days (∼150 days), though the sources of smoke in these regions differ. In the Western CONUS and SC, the primary source of smoke is wildfires. Conversely, in the central-south and southeastern regions of the CONUS, prescribed fires are the main source of smoke.^67^ The northeastern regions of the study domain are less frequently impacted by smoke (∼100 days) but can still experience significant smoke pollution from long-range transport.^68,69^

We summarized the background, total, and smoke-specific concentrations of PM_2.5_ OC and EC across different time scales and climate regions in Figure S8. From 2002 to 2019, both the CONUS and SC experienced a declining trend in background carbonaceous PM_2.5,_ with annual concentrations falling below 0.95 μg/m^3^ for OC and 0.20 μg/m^3^ for EC by 2019. When comparing the background carbonaceous PM_2.5_ between the periods 2002–2010 and 2011–2019, improvements in annual background PM_2.5_ were observed, with reductions of 0.27 μg/m^3^ for the CONUS and 0.17 μg/m^3^ for SC after 2011. The CONUS climate regions of Southeast, South, Central, and Northeast exhibited higher background concentrations and more significant reductions over the years (Figure 3). Additionally, urban areas displayed higher background concentrations of carbonaceous PM_2.5_ (Figure S9), with long-term average concentrations reaching approximately 2 μg/m^3^ for OC and 1 μg/m^3^ for EC. Elevated background PM_2.5_ OC levels were also common in many rural and forested areas of the South, Central and Southeast CONUS climate regions, while high levels of PM_2.5_ EC were mostly concentrated in urban centers across the study domain.

**Figure 3:**
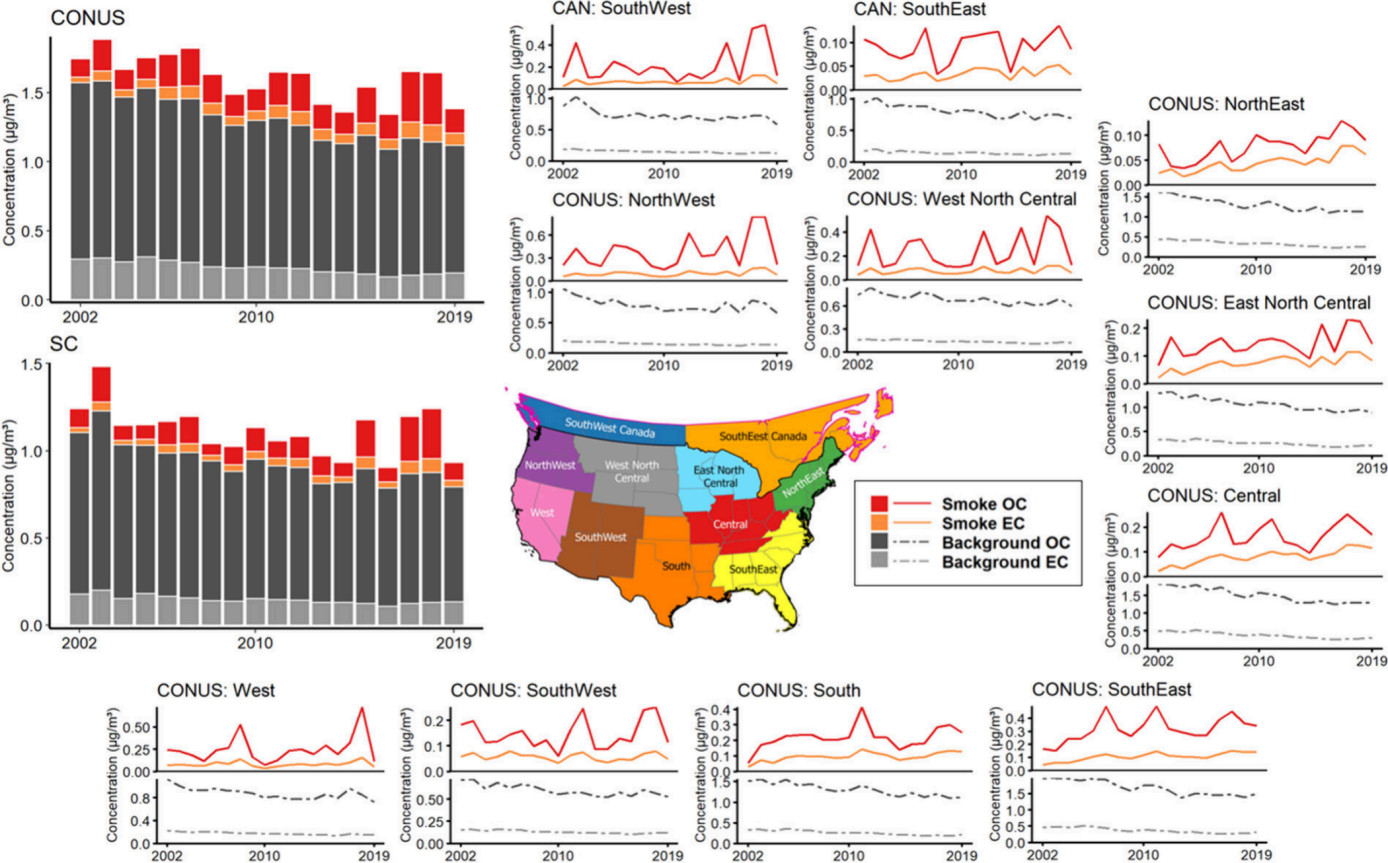
Annual average concentration of background and smoke PM_2.5_ OC and EC in the CONUS and SC, categorized by climate regions and spanning the study period from 2002 to 2019.

Considering the impacts of fire smoke, we observed a distinct contribution of smoke PM_2.5_ OC, whereas the contribution of smoke PM_2.5_ EC was smaller and showed less fluctuation over the years. The mean annual concentration of smoke PM_2.5_ OC was 0.22 μg/m^3^ for the CONUS and 0.14 μg/m^3^ for SC, while smoke EC concentrations were 0.08 μg/m^3^ for the CONUS and 0.05 μg/m^3^ for SC. Combining smoke PM_2.5_ OC and EC, smoke carbonaceous PM_2.5_ accounted for 19 and 16% of the total concentrations in the CONUS and SC, respectively. At the monthly level, smoke carbonaceous PM_2.5_ exhibited a notable increase during the peak months of wildfire season (July–November) (Figure S10), with average monthly concentrations rising to 2.38 μg/m^3^ for the CONUS and 2.60 μg/m^3^ for SC, accounting for 58 and 57% of total concentrations, respectively.

The Western and Southern CONUS climate regions (i.e., Northwest, West, West North Central, South, and Southeast) and the Southwestern Canada experienced more fire smoke impacts, including wildland fire and prescribed fire, resulting in increased smoke carbonaceous PM_2.5_ at both regional and national scales (Figure 3). Additionally, the annual mean concentrations of smoke carbonaceous PM_2.5_ increased by 0.08 μg/m^3^ for the CONUS and 0.05 μg/m^3^ for SC before and after 2011. This intensifying trend in fire smoke has offset nearly one-third of the improvements in background concentrations. Megafire years of 2012, 2015, 2017, and 2018 experienced significantly higher concentrations of both smoke PM_2.5_ OC and EC at the monthly and annual levels.

Our 1 × 1 km^2^ prediction maps revealed different spatial distributions between long-term smoke OC and EC concentrations (Figure S9). High smoke OC concentrations (>0.50 μg/m^3^) were primarily observed in rural areas of the Western CONUS and SC, and were sporadically distributed in the Southeastern CONUS. Elevated EC concentrations (>0.15 μg/m^3^) were often collocated with high smoke OC, with the Southeastern CONUS, particularly Georgia, Florida, Mississippi, and Texas, exhibiting higher smoke EC concentrations (>0.20 μg/m^3^). Long-term average concentrations of smoke carbonaceous PM_2.5_ were highest in the Western CONUS and Southwestern Canada, particularly in California, Idaho, and Montana (Figure 4). The Southeastern CONUS also experienced comparable concentrations of smoke carbonaceous PM_2.5,_ with urban centers such as Charlotte and Atlanta, and their surrounding areas, showing higher exposure levels than urban areas in other parts of the study domain (Figure 4). During megafire years (2012, 2015, 2017, and 2018), elevated smoke carbonaceous PM_2.5_ was concentrated in rural regions of the Western CONUS, as well as urban centers such as Los Angeles, San Francisco, Seattle and Vancouver, as captured in the annual prediction maps (Figures S11 and S12). However, long-term averages inevitably mask the short-lived, often distinctly shaped smoke plumes that represent individual fire events. To demonstrate the model’s ability in capturing localized event-scale peaks, we mapped smoke carbonaceous PM_2.5_ for an extreme smoke day on August 23, 2018 (Figure S13), when extensive wildfires in western Canada and the western CONUS affected both coastal cities nearby and distant downwind cities.^70^ The western cities (i.e., San Francisco, Seattle, and Vancouver) displayed consistent extreme pollution level, reflecting the dense, freshly emitted plume. After long-range transport, the plume reached southeastern cities (i.e., Charlotte and Atlanta) in a considerably diluted state, producing a broad but moderate concentration increase. Los Angeles, located near fire sources but did not experience direct and strong smoke impacts, showed clear intraurban variability, potentially due to the topography and landcover heterogeneity.

**Figure 4:**
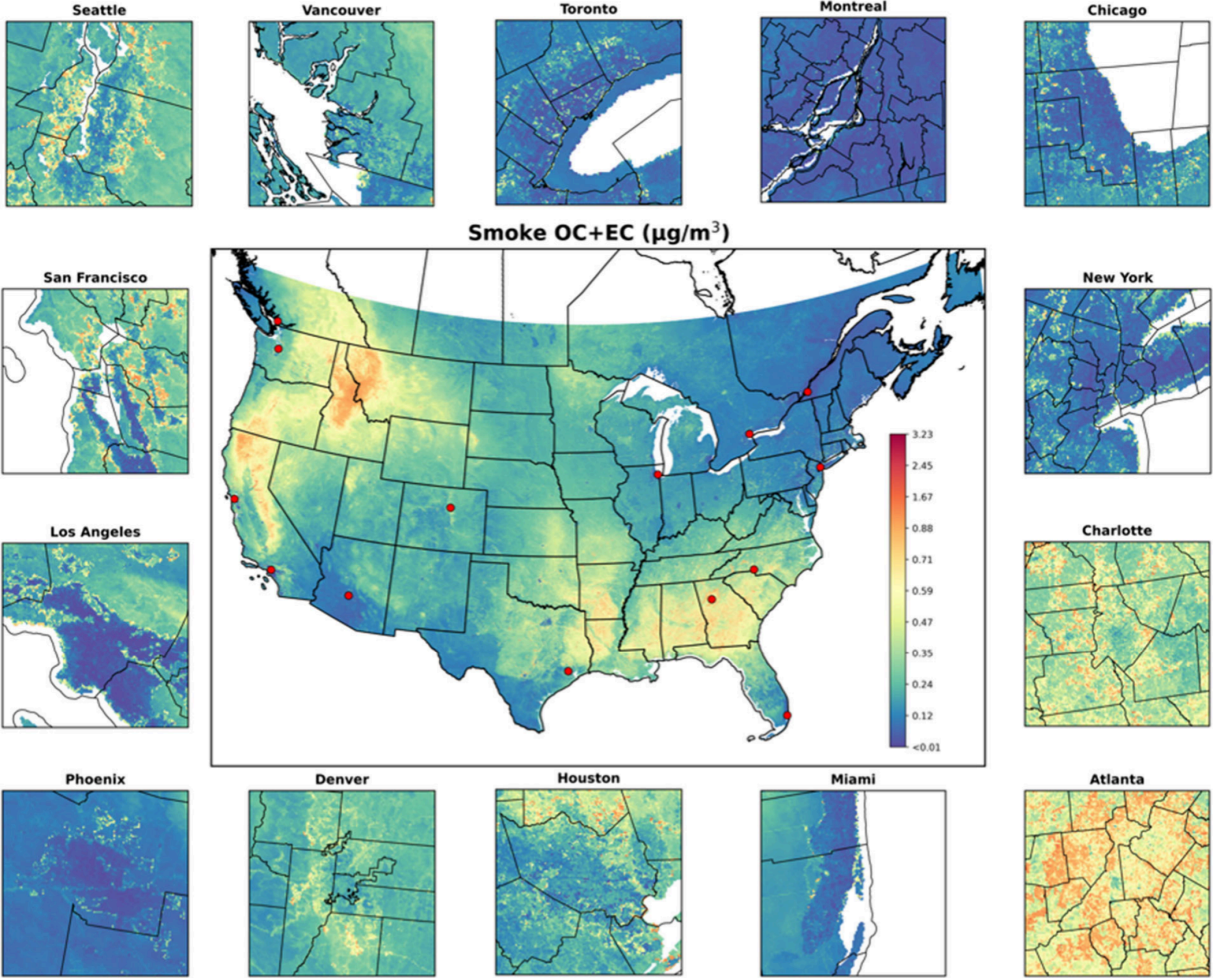
Long-term average annual concentration of smoke carbonaceous PM_2.5_ from 2002 to 2019, with major urban centers (located at red points) zoomed in for detailed visualization.

### Increasing Impact of Wildland Fire Smoke on Air Quality and Population Exposure

We evaluated the populations affected by heavy fire smoke in the CONUS and SC. A “heavy fire smoke grid day” was defined as a grid day when the total carbonaceous PM_2.5_ concentration exceeded 1 μg/m^3^ and the smoke carbonaceous PM_2.5_ constituted more than 50% of the total concentration. We compared the cumulative daily populations affected by heavy fire smoke during the periods 2002–2010, 2011–2019, and specifically the five years from 2015–2019 (Figure 5). Our analysis revealed a clear increasing trend in population exposed over the years, corresponding to the broadening of wildfire smoke impacts. During 2002–2010, there was an average of 467 million person days of exposure to heavy fire smoke prior to July first, while this number rose by 70% to 793 million person days during 2011–2019. By year’s end, cumulative exposure increased from 986 million person days in 2002–2010 to 1.8 billion person days in 2011–2019, representing an 83% increase. To account for the influence of population growth on these trends, we also calculated the annual exposure days per capita. On average, individuals experienced 3.0 days of heavy smoke exposure per year in 2002–2010, which increased to 5.1 days per year in 2011–2019. The megafire years of 2017 and 2018 had particularly severe impacts, with cumulative exposure exceeding 3 billion person days (Figure 5), equivalent to 9.0 exposure days per person per year. Separate figures for cumulative person days of exposure to heavy fire smoke in the CONUS and SC across years are provided in Figure S14.

**Figure 5:**
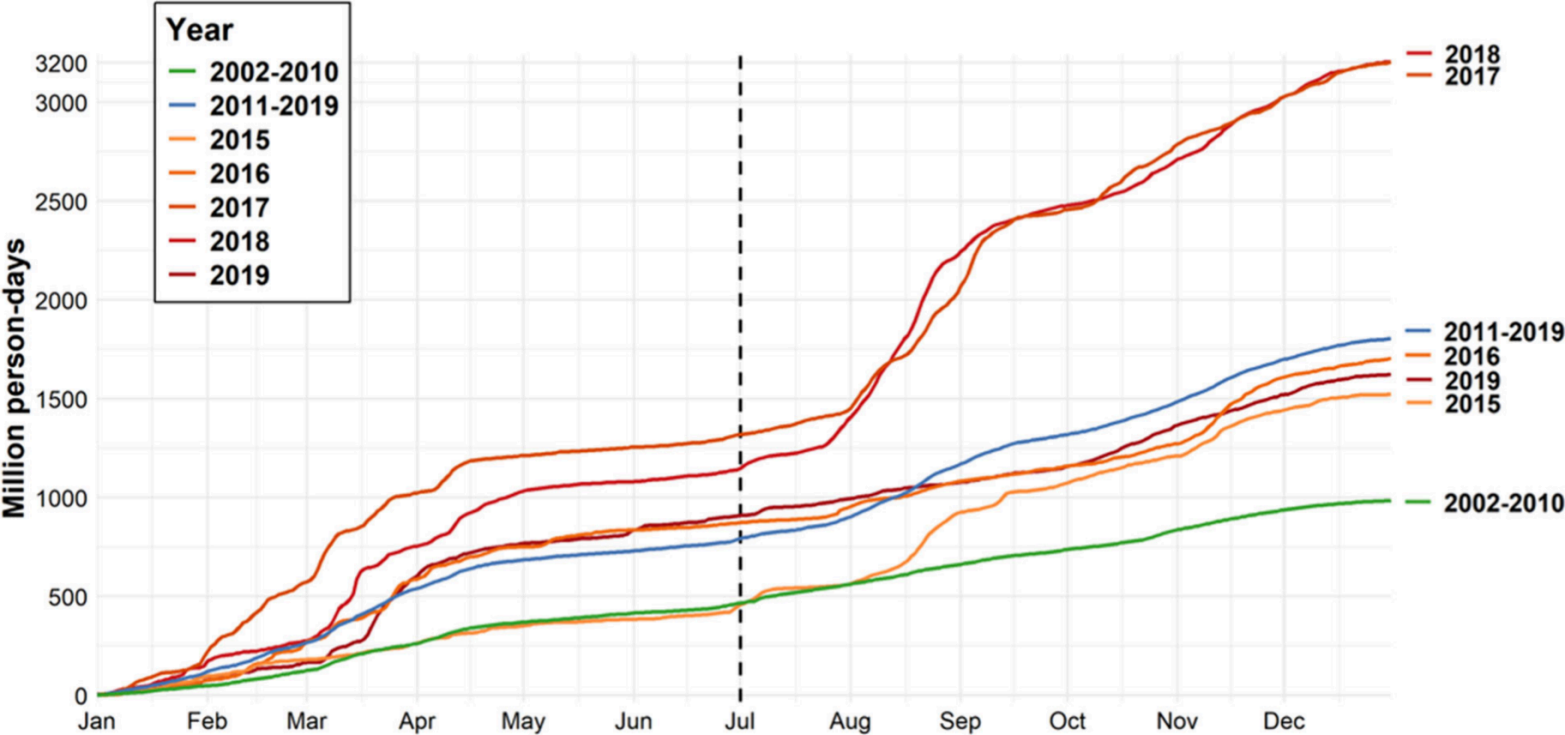
Cumulative person days exposed to heavy fire smoke in the CONUS and SC (unit: million person day). Note: A person day is defined as one individual exposed to heavy fire smoke for 1 day.

### Excess Mortality Due to Smoke Exposure

The annual non-accidental mortality rate and total deaths attributable to smoke carbonaceous PM_2.5_ from 2003 to 2020 are mapped at the county level in the CONUS and the census division (CD) level in SC (Figure S15). Consistent with the spatial distribution of high smoke carbonaceous PM_2.5_ concentrations in the Western and Southeastern CONUS and Southwestern Canada, counties and CDs in these areas exhibited elevated annual mortality rates, exceeding 3 deaths per 100 000 people (Figure S15A). The CONUS counties with higher annual death counts (>3 deaths per year) were generally located in California, Oregon, Washington, Florida, Georgia, South Carolina, North Carolina, and Texas (Figure S15B), areas characterized by high population density, frequent smoke impact and elevated smoke carbonaceous PM_2.5_. On average, 7455 (95% CI: 6058, 8852) non-accidental deaths per year in the CONUS were attributable to long-term exposure to fire smoke carbonaceous PM_2.5_ (Table S14), with the southeastern CONUS contributing 4698 (95% CI: 3859, 5550) deaths per year (63.0%), and the western region contributing 1,567 (95% CI: 1254, 1881) deaths per year (21.0%).

For sensitivity analyses, when smoke carbonaceous PM_2.5_ concentrations were rescaled to represent 50–95% of the total smoke PM_2.5_ mass, the mean annual deaths in the CONUS ranged from 7751 (95% assumption, Table S15) to 11 160 (50% assumption). During the megafire year of 2018, the corresponding interval was 12 059–15 117 deaths. The second sensitivity analysis yielded a higher estimate of 24 671 annual deaths (Table S15) on average, with a maximum of 32 292 deaths in 2018.

In SC, higher annual deaths were observed in urban areas of British Columbia, Ontario, and Quebec (Figure S15B), with an average of 259 (95% CI: 136, 383) non-accidental deaths per year (Table S17). Annual total deaths in the southeastern region remained relatively stable over the study period (Figure 6), whereas deaths in the western and northeastern regions, as well as in SC, fluctuated significantly in response to variations in wildfire intensity.

**Figure 6:**
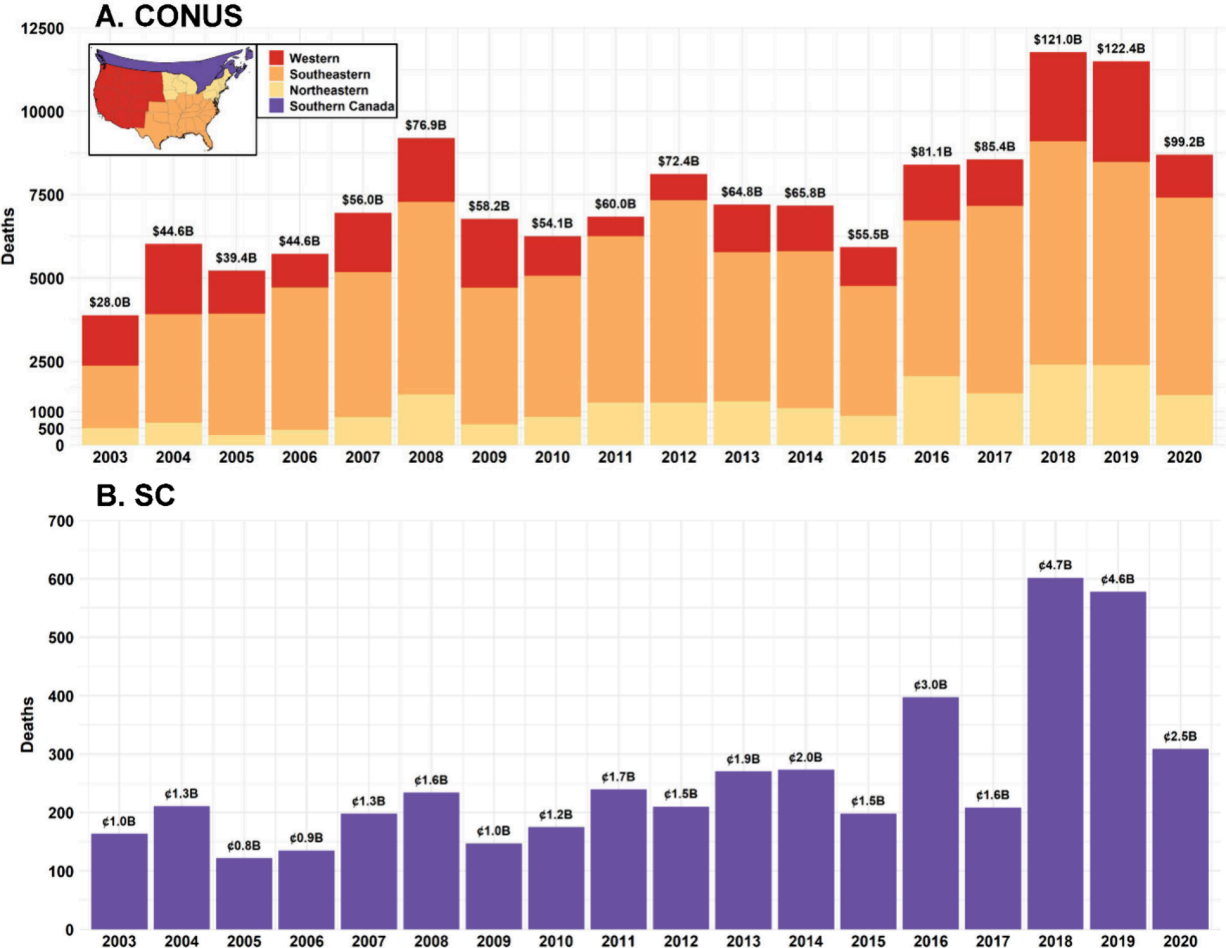
Annual total deaths attributable to fire smoke carbonaceous PM_2.5_ in the CONUS (subplot A) and SC (subplot B). Note: Climate regions in the CONUS were grouped into three major regions based on the U.S. National Prescribed Fire Use Survey Report and our analysis of smoke-impacted areas (see Figure S7). These regions are defined as follows: the western region (i.e., climate regions: West, Southwest, Northwest, and West North Central), the southeastern region (i.e., climate regions: Southeast, Central, and South), and the northeastern region (i.e., climate regions: Northeast and East North Central). The annual total monetized damages for the CONUS and SC are labeled at the top of each column (units: billion USD for the CONUS and billion CAD for SC).

The average monetized damages associated with these mortality estimates were approximately 68.3 (95% CI: 31.9, 104.0) billion USD per year for the CONUS and 1.9 (95% CI: 1.0, 2.8) billion CAD per year for SC (Tables S16 and S17). The southeastern region contributed 43.0 (95% CI: 20.0, 65.4) billion USD annually, and the western region contributed 14.2 (95% CI: 6.6, 21.6) billion USD. In 2018 and 2019, both mortality and monetized damages nearly doubled compared to the average levels, leading to monetized damages exceeding 120 billion USD for the CONUS and 4.6 billion CAD for SC (Figure 6).

## Discussion

To the best of our knowledge, this is the first study to model the full-coverage concentration of fire smoke-derived carbonaceous PM_2.5_ with high spatial and temporal resolution across both the CONUS and SC. We developed a three-level RASL framework to estimate both daily and long-term smoke carbonaceous PM_2.5_ from 2002 to 2019. Our analysis identified frequent smoke impact and elevated concentrations of smoke-derived carbonaceous PM_2.5_ in the Western and Southeastern CONUS, as well as in SC. Over the past decade, wildfire seasons have started earlier, lasted longer, and wildfire activity has intensified, resulting in increased population exposure to wildfire smoke. Specifically, population exposure to heavy smoke increased from an average of 3.0 days per year in 2002–2010 to 5.1 days per year in 2011–2019, driven largely by climate change which has created more favorable conditions for wildfires.^71–76^ We estimated that long-term exposure to smoke carbonaceous PM_2.5_ resulted in an average of 7455 and 259 non-accidental deaths per year in the CONUS and SC, respectively, with the Southeastern CONUS contributing the most deaths. Recent megafire years (e.g., 2017 and 2018) exhibited extremely high concentrations of carbonaceous PM_2.5_ and corresponding health burdens. Without significant mitigation efforts, future climate models predict an alarming increase in wildfire frequency and severity, which poses further risks to ecosystems, air quality, and public health.^77,78^

The long-term concentration of PM_2.5_ mass from fire smoke in the CONUS and Canada has been examined in previous literature. Our findings on the spatial and temporal patterns of smoke carbonaceous PM_2.5_ are consistent with these prior studies. For example, Childs et al. estimated smoke PM_2.5_ over the CONUS at a 10 km resolution and observed increased smoke pollution and smoke-impacted days over the past decade, especially in the Western U.S. and in the years 2017 and 2018.^56^ Alpizar et al. conducted a multiyear analysis of the FireWork outputs from May to September to quantify wildfire contribution to total PM_2.5_ across North America.^79^ FireWork is an online meteorology-chemistry model developed by Environment and Climate Change Canada, providing near-real-time forecasts of biomass burning PM_2.5_ across North America.^80^ The findings showed that most wildfire events were concentrated in the Western CONUS, as well as in Western, Northern, and Central Canada. While previous studies have offered valuable insights into wildfire smoke PM_2.5_, our approach distinguishes between smoke PM_2.5_ OC and EC, allowing further study on smoke composition and potential toxicity that may result in different health impacts. Moreover, our daily estimates at 1 × 1 km^2^ resolution better captures local hotspots and intraurban gradients during extreme-smoke days and allow for more precise long-term estimates, which is essential for reducing exposure misclassification in further analyses.

Additionally, our results indicated a significant intensification of wildfires with higher concentrations of smoke carbonaceous PM_2.5_ across our study period, which has offset nearly one-third of the improvements in background air quality across the CONUS and SC, largely due to efforts such as the Clean Air Act.^81^ This intensifying trend in wildfire smoke stagnated or even reversed the declining trend of background concentrations in most regions. Our findings align with existing literature on smoke PM_2.5_ mass. Burke et al. reported that areas affected by wildfire smoke have doubled over the past two decades in the CONUS, and that wildfire smoke has influenced the average annual PM_2.5_ trend in 41 out of 48 CONUS States since 2016.^4,82^ Notably, smoke has offset approximately 25% of the overall improvement in air quality. As studies of Canadian wildfire-smoke impacts remain relatively scarce, our findings add to this body of literature by characterizing smoke PM_2.5_ patterns in SC and transboundary transport. We observed that the western provinces can be a major source region for downwind smoke, which also affects air quality in the eastern provinces and the North-Central and Northeastern U.S. In recent decades, wildfires are becoming both more common and more destructive in Canada, extending from British Columbia and Alberta to the Atlantic region.^83,84^ The Canada’s record-breaking wildfires in 2023 demonstrated that these plumes can cross the North American continent and even the Atlantic Ocean.^85^ Carter et al. showed that boreal fire emissions propagated throughout North America, influencing PM_2.5_ as far south as the U.S. Midwest and Atlantic seaboard.^86^ Such patterns highlight how the entire North American continent is interconnected by large-scale fire activity and regional meteorology. Future research that encompassing Alaska, Northern Canada, and Mexico with high spatiotemporal resolution is needed to achieve a more comprehensive understanding of the wildfire smoke activity across North America.

Prescribed fires are widely recognized as one of the most effective ways to prevent potential wildfires and sustain biodiversity in all regions beyond the Southeastern CONUS.^87–89^ Our study revealed lower concentrations of smoke carbonaceous PM_2.5_ in the Southeastern CONUS during megafire years compared to the Western CONUS. However, our analysis also indicates that prescribed fires do not necessarily translate into better air quality. They instead lead to consistent smoke pollution with elevated carbonaceous PM_2.5_ concentration over time that cause adverse health effects from long-term exposure, which has not been extensively discussed in prior fire smoke modeling studies or government data sets. In the Southeastern CONUS region, where prescribed fires are the primary sources of smoke, we estimated 4702 attributable deaths per year, which is higher than the combined deaths in the Western CONUS (1,568 deaths per year) and Northeastern CONUS (1192 deaths per year) regions. Several factors contribute to this outcome. First, unlike the Western U.S., where wildfires occur sporadically but at high intensity, the Southeastern CONUS experiences regular smoke pollution from frequent prescribed fires. Our results indicated that annual regional average concentrations of smoke carbonaceous PM_2.5_ in the Southeast CONUS are comparable to those in the Western CONUS (∼0.4 μg/m^3^). While prescribed fires effectively reduce risks of wildfires, they also result in frequent and localized smoke pollution in the Southeastern CONUS, where prescribed burns are conducted throughout the year.^67^ Second, prescribed fire smoke frequently affects densely populated urban and suburban areas in the Southeast CONUS, such as Atlanta and Charlotte, even though the fires are smaller and controlled.^67^ In contrast, wildfires in the Western CONUS typically occur in more remote, forested areas, such as the Cascades and Rocky Mountains. While wildfire smoke can travel long distances to urban centers like Los Angeles, San Francisco, and even the Northeastern CONUS, the primary impact is often concentrated in less densely populated areas. Third, residents of Southeastern states such as Georgia and Florida are more accustomed to prescribed fires.^90^ Engebretson et al. reported that Southern state residents demonstrate significantly higher tolerance of potential health impacts from prescribed fires compared to those in Western states.^91^ While this acceptance reduces social barriers to the use of prescribed fires, it also lowers public vigilance regarding exposure to fire smoke pollutants. In contrast, the perception of wildfire risk in the Western U.S. has been heightened by the prevalence of megafires and media coverage, leading to a higher public awareness of the dangers posed by wildfire smoke and harm-reduction behaviors.

The average monetized damages associated with attributable deaths in the Southeastern U.S. is 43.0 billion USD per year, and this cost for the CONUS has exceeded 120 billion USD in recent megafire years. In contrast, the U.S. allocated $1.73 billion to wildland fire management in 2024, with $214.5 million dedicated to fuels management.^92^ Considerable evidence in the scientific literature supports prescribed fire as a cost-effective method for mitigating wildfire risk and reducing carbon emissions.^93–95^ However, most of these studies overlook the significant health impacts associated with smoke exposure from prescribed fire, which can be transported to nearby populated areas. When considering health-related costs, the cost-effectiveness of prescribed burns is called into question, as these fires can cause damage over 200 times greater than the budget. To better inform policy, it is crucial to develop a more accurate and comprehensive cost-benefit assessment for prescribed burns by incorporating health-related monetized damages from smoke exposure. Additionally, policies should prioritize minimizing human smoke exposure by improving monitoring networks of both PM_2.5_ mass and carbonaceous components and preparing communities for potential health impacts. Enhancing communication strategies to warn residents and provide resources, such as air quality alerts and protective equipment, should be an essential component of these policies. Beyond policy improvements, a more sophisticated prescribed fire management system is necessary, one that considers the conditions of each fire, including risk factors such as weather and proximity to populations, and the long-term benefits of prescribed burns in limiting wildfires impacts.^96^ Achieving this balance between prescribed fire and public health is essential to ensure that prescribed burns remain a valuable tool for ecosystem health and wildfire prevention.

Our study has several implications. First, it provides a high-resolution fire smoke product with full coverage for the CONUS and SC, offering insights into the occurrence and distribution of fire smoke impacts, and quantitative estimates of background and smoke carbonaceous PM_2.5_ concentrations. The comprehensive spatial and temporal coverage of our predictions enables future research on the health and environmental impacts of exposure to altered PM_2.5_ composition by fire smoke. Second, our findings suggested that wildland fires have intensified over the past decade, leading to an increase in deaths associated with long-term exposure to smoke carbonaceous PM_2.5_. Finally, shortcomings were identified in the current prescribed fire permit databases because some states in the Southeastern U.S. (e.g., Texas, Arkansas, and Missouri) do not require prescribed burn permits and instead rely on voluntary reporting, resulting in incomplete records and potential underestimation of prescribed fire activity.^67^ By combining the prescribed fire permit databases with our study’s high-resolution smoke predictions, future efforts could better track prescribed fire activities in terms of locations, durations, sizes and transmissions. As climate change continues to challenge wildfire risk mitigation and biodiversity conservation, our study could inform the incorporation of potential health impacts in the cost-benefit analyses of prescribed fire policies and management tools.

Several limitations of our study should be noted. First, the level 3 monthly GAM residual adjustment relies on information from ground-based monitors. The corrective performance is constrained in unpopulated regions with sparse monitoring network, particularly the Central U.S. and Northern Canada. Expanding monitoring coverage in rural areas would help refine the model and better reflect the impact of fire smoke. Second, there is currently no research specifically investigating the mortality attributable to smoke carbonaceous PM_2.5_. As a result, our study relies on mortality risk estimates based on total smoke PM_2.5_. Because OC and EC constitute only a fraction of total smoke PM_2.5_, their concentrations are systematically lower and assigned to lower concentration bins specified by Ma et al. The misclassification, together with the possibility that carbonaceous species possess greater toxicity than total smoke PM_2.5_, may lead to an underestimation of non-accidental deaths and monetized damages attributable to smoke carbonaceous PM_2.5_. Our sensitivity analyses illustrated this possibility. The upper bound estimate was approximate 8000–11 000 annual deaths if considering different carbonaceous fractions of total smoke, while the estimate was substantially higher (∼25 000) when coefficients from PM_2.5_ speciation-based epidemiological study were applied. Future research focusing on the mortality risk associated with specific components of smoke PM_2.5_ is needed to more accurately estimate the impact of carbonaceous PM_2.5_ from fire smoke. Third, the annual smoke-mortality relationships for both the CONUS and SC applied in our study may not be entirely applicable to the 2020 baseline mortality rate, which was impacted by the COVID pandemic. Fourth, our modeling domain excludes Alaska and the northern Canadian territories, where boreal wildfires are frequent and intense.^97^ By omitting these high-latitude source regions we likely underestimate both the magnitude and spatial transport of transboundary smoke transport.

In conclusion, our study highlights the growing impact of fire smoke carbonaceous PM_2.5_ and its adverse effects on public health across the CONUS and SC. With wildfires intensifying and becoming more frequent due to climate change, our findings underscore the urgent need for comprehensive prescribed fire management strategies that balance ecological benefits with the reduction of smoke-related health risks. This work provides a valuable foundation for future research and policymaking to address the dual challenges of wildfire prevention and public health protection in an increasingly fire-prone environment.

## Supplementary Material



## Notes

Materials and methods for auxiliary predictor processing, modeling framework, and model performance evaluation, sensitivity analyses for CONUS mortality burden (sections S1–S4), supplemental tables (Tables S1–S17), and supplemental figures (Figures S1–S15) (PDF)

## References

[1] Fernandez-Anez N., Krasovskiy A., Müller M., Vacik H., Baetens J., Hukić E., Kapovic Solomun M., Atanassova I., Glushkova M., Bogunović I. (2021). Current wildland fire
patterns and challenges in Europe: A synthesis of national perspectives. Air, Soil and Water Research.

[2] Senande-Rivera M., Insua-Costa D., Miguez-Macho G. (2022). Spatial and temporal expansion of
global wildland fire activity in response to climate change. Nat. Commun..

[3] Abatzoglou J. T., Williams A. P. (2016). Impact of anthropogenic climate change
on wildfire
across western US forests. Proc. Natl. Acad.
Sci. U. S. A..

[4] Burke M., Driscoll A., Heft-Neal S., Xue J., Burney J., Wara M. (2021). The changing risk and burden of wildfire
in the United States. Proc. Natl. Acad. Sci.
U. S. A..

[5] Fernandes P. M. (2015). Empirical
support for the use of prescribed burning as a fuel treatment. Current Forestry Reports.

[6] Fowler C., Konopik E. (2007). The history of fire
in the southern United States. Human Ecology
Review.

[7] Melvin, M. 2018 National Prescribed Fire Use Survey Report; Coalition of Prescribed Fire Councils, Inc.: Newton, GA, 2018.

[8] Brey S. J., Barnes E. A., Pierce J. R., Wiedinmyer C., Fischer E. V. (2018). Environmental conditions, ignition
type, and air quality
impacts of wildfires in the southeastern and western United States. Earth’s Future.

[9] Haikerwal A., Reisen F., Sim M. R., Abramson M. J., Meyer C. P., Johnston F. H., Dennekamp M. (2015). Impact of
smoke from prescribed burning:
Is it a public health concern?. J. Air Waste
Manage. Assoc..

[10] Jaffe D. A., O’Neill S. M., Larkin N. K., Holder A. L., Peterson D. L., Halofsky J. E., Rappold A. G. (2020). Wildfire and prescribed
burning impacts
on air quality in the United States. J. Air
Waste Manage. Assoc..

[11] Prunicki M., Kelsey R., Lee J., Zhou X., Smith E., Haddad F., Wu J., Nadeau K. (2019). The impact
of prescribed
fire versus wildfire on the immune and cardiovascular systems of children. Allergy.

[12] Xu R., Yu P., Abramson M. J., Johnston F. H., Samet J. M., Bell M. L., Haines A., Ebi K. L., Li S., Guo Y. (2020). Wildfires,
Global Climate Change, and Human Health. New
England Journal of Medicine.

[13] Jaffe D., Hafner W., Chand D., Westerling A., Spracklen D. (2008). Interannual variations in PM_2.5_ due to wildfires
in the Western United States. Environ. Sci.
Technol..

[14] Sullivan A., Holden A., Patterson L., McMeeking G., Kreidenweis S., Malm W., Hao W., Wold C., Collett J. (2008). A method for smoke marker measurements
and its potential application for determining the contribution of
biomass burning from wildfires and prescribed fires to ambient PM_2.5_ organic carbon. Journal of Geophysical
Research: Atmospheres.

[15] Zhang Q., Jimenez J. L., Canagaratna M., Allan J. D., Coe H., Ulbrich I., Alfarra M., Takami A., Middlebrook A., Sun Y. (2007). Ubiquity and dominance of oxygenated species in organic aerosols
in anthropogenically-influenced Northern Hemisphere midlatitudes. Geophysical research letters.

[16] Bond T. C., Doherty S. J., Fahey D. W., Forster P. M., Berntsen T., DeAngelo B. J., Flanner M. G., Ghan S., Kärcher B., Koch D. (2013). Bounding
the role of black carbon in the climate system:
A scientific assessment. Journal of geophysical
research: Atmospheres.

[17] Orellano P., Reynoso J., Quaranta N., Bardach A., Ciapponi A. (2020). Short-term
exposure to particulate matter (PM_10_ and PM_2.5_), nitrogen dioxide (NO_2_), and ozone (O_3_) and
all-cause and cause-specific mortality: Systematic review and meta-analysis. Environ. Int..

[18] Zhang L., Wilson J. P., Zhao N., Zhang W., Wu Y. (2022). The dynamics
of cardiovascular and respiratory deaths attributed to long-term PM_2.5_ exposures in global megacities. Science
of The Total Environment.

[19] Pun V. C., Kazemiparkouhi F., Manjourides J., Suh H. H. (2017). Long-term PM_2.5_ exposure
and respiratory, cancer, and cardiovascular mortality
in older US adults. American journal of Epidemiology.

[20] Li Z., Tang Y., Song X., Lazar L., Li Z., Zhao J. (2019). Impact of ambient PM_2.5_ on adverse birth outcome and potential
molecular mechanism. Ecotoxicology and Environmental
Safety.

[21] Ma Y., Zang E., Liu Y., Wei J., Lu Y., Krumholz H. M., Bell M. L., Chen K. (2024). Long-term exposure
to wildland fire smoke PM_2.5_ and mortality in the contiguous
United States. Proc. Natl. Acad. Sci. U. S.
A..

[22] Ye T., Xu R., Yue X., Chen G., Yu P., Coêlho M. S. Z. S., Saldiva P. H. N., Abramson M. J., Guo Y., Li S. (2022). Short-term exposure to wildfire-related PM_2.5_ increases
mortality risks and burdens in Brazil. Nat.
Commun..

[23] Eisenman D. P., Galway L. P. (2022). The mental health and well-being effects of wildfire
smoke: a scoping review. BMC public health.

[24] Karanasiou A., Alastuey A., Amato F., Renzi M., Stafoggia M., Tobias A., Reche C., Forastiere F., Gumy S., Mudu P., Querol X. (2021). Short-term
health effects
from outdoor exposure to biomass burning emissions: A review. Science of The Total Environment.

[25] Zhu Q., Zhang D., Wang W., D’Souza R. R., Zhang H., Yang B., Steenland K., Scovronick N., Ebelt S., Chang H. H., Liu Y. (2024). Wildfires
are associated with increased emergency department visits for anxiety
disorders in the western United States. Nature
Mental Health.

[26] Danesh
Yazdi M., Amini H., Wei Y., Castro E., Shi L., Schwartz J. D. (2024). Long-term exposure to PM_2.5_ species and
all-cause mortality among Medicare patients using mixtures analyses. Environmental Research.

[27] Hvidtfeldt U. A., Geels C., Sørensen M., Ketzel M., Khan J., Tjønneland A., Christensen J. H., Brandt J., Raaschou-Nielsen O. (2019). Long-term
residential exposure to PM_2.5_ constituents and mortality
in a Danish cohort. Environ. Int..

[28] Wang Y., Xiao S., Zhang Y., Chang H., Martin R. V., Van Donkelaar A., Gaskins A., Liu Y., Liu P., Shi L. (2022). Long-term
exposure to PM_2.5_ major components and mortality
in the southeastern United States. Environ.
Int..

[29] Chen J., Li C., Ristovski Z., Milic A., Gu Y., Islam M. S., Wang S., Hao J., Zhang H., He C., Guo H., Fu H., Miljevic B., Morawska L., Thai P., Lam Y. F., Pereira G., Ding A., Huang X., Dumka U. C. (2017). A review
of biomass burning: Emissions and impacts
on air quality, health and climate in China. Science of The Total Environment.

[30] Chen H., Samet J. M., Bromberg P. A., Tong H. (2021). Cardiovascular health
impacts of wildfire smoke exposure. Particle
and Fibre Toxicology.

[31] Azevedo J. M., Gonçalves F. L.
T., de Fátima
Andrade M. (2011). Long-range
ozone transport and its impact on respiratory and cardiovascular health
in the north of Portugal. International Journal
of Biometeorology.

[32] Marlier M. E., DeFries R. S., Voulgarakis A., Kinney P. L., Randerson J. T., Shindell D. T., Chen Y., Faluvegi G. (2013). El Niño and
health risks from landscape fire emissions in southeast Asia. Nature Climate Change.

[33] Reid C. E., Brauer M., Johnston F. H., Jerrett M., Balmes J. R., Elliott C. T. (2016). Critical Review
of Health Impacts of Wildfire Smoke
Exposure. Environ. Health Perspect..

[34] Kiser D., Metcalf W. J., Elhanan G., Schnieder B., Schlauch K., Joros A., Petersen C., Grzymski J. (2020). Particulate
matter and emergency visits for asthma: a time-series study of their
association in the presence and absence of wildfire smoke in Reno,
Nevada, 2013–2018. Environmental Health.

[35] Liu J. C., Wilson A., Mickley L. J., Dominici F., Ebisu K., Wang Y., Sulprizio M. P., Peng R. D., Yue X., Son J.-Y., Anderson G. B., Bell M. L. (2017). Wildfire-specific
fine particulate matter and risk of hospital admissions in urban and
rural counties. Epidemiology.

[36] Frohn L. M., Geels C., Andersen C., Andersson C., Bennet C., Christensen J. H., Im U., Karvosenoja N., Kindler P. A., Kukkonen J., Lopez-Aparicio S., Nielsen O.-K., Palamarchuk Y., Paunu V.-V., Plejdrup M. S., Segersson D., Sofiev M., Brandt J. (2022). Evaluation of multidecadal
high-resolution atmospheric chemistry-transport modelling for exposure
assessments in the continental Nordic countries. Atmos. Environ..

[37] Cleland S. E., West J. J., Jia Y., Reid S., Raffuse S., O’Neill S., Serre M. L. (2020). Estimating wildfire smoke concentrations
during the October 2017 California fires through BME space/time data
fusion of observed, modeled, and satellite-derived PM_2.5_. Environ. Sci. Technol..

[38] Zhang D., Wang W., Xi Y., Bi J., Hang Y., Zhu Q., Pu Q., Chang H., Liu Y. (2023). Wildland Fires Worsened
Population Exposure to PM_2.5_ Pollution in the Contiguous
United States. Environ. Sci. Technol..

[39] Li Y., Tong D., Ma S., Zhang X., Kondragunta S., Li F., Saylor R. (2021). Dominance
of wildfires impact on air quality exceedances
during the 2020 record-breaking wildfire season in the United States. Geophys. Res. Lett..

[40] Pan X., Ichoku C., Chin M., Bian H., Darmenov A., Colarco P., Ellison L., Kucsera T., da Silva A., Wang J., Oda T., Cui G. (2020). Six global biomass
burning emission datasets: intercomparison and application in one
global aerosol model. Atmospheric Chemistry
and Physics.

[41] Jin Z., Pu Q., Janechek N., Zhang H., Wang J., Chang H., Liu Y. (2024). A MAIA-like modeling framework to estimate PM_2.5_ mass
and speciation concentrations with uncertainty. Remote Sensing of Environment.

[42] Van
der Laan M. J., Polley E. C., Hubbard A. E. (2007). Super learner. Statistical applications in genetics and molecular biology.

[43] Di Q., Amini H., Shi L., Kloog I., Silvern R., Kelly J., Sabath M. B., Choirat C., Koutrakis P., Lyapustin A., Wang Y., Mickley L. J., Schwartz J. (2019). An ensemble-based
model of PM_2.5_ concentration across the contiguous United
States with high spatiotemporal resolution. Environ. Int..

[44] Solomon P. A., Crumpler D., Flanagan J. B., Jayanty R., Rickman E. E., McDade C. E. (2014). US national PM_2.5_ chemical speciation monitoring
networks—CSN and IMPROVE: description of networks. J. Air Waste Manage. Assoc..

[45] Hansen D. A., Edgerton E. S., Hartsell B. E., Jansen J. J., Kandasamy N., Hidy G. M., Blanchard C. L. (2003). The southeastern
aerosol research
and characterization study: Part 1—Overview. J. Air Waste Manage. Assoc..

[46] Dabek-Zlotorzynska E., Dann T. F., Martinelango P. K., Celo V., Brook J. R., Mathieu D., Ding L., Austin C. C. (2011). Canadian National
Air Pollution Surveillance (NAPS) PM_2.5_ speciation program:
Methodology and PM_2.5_ chemical composition for the years
2003–2008. Atmos. Environ..

[47] Appel K. W., Bash J. O., Fahey K. M., Foley K. M., Gilliam R. C., Hogrefe C., Hutzell W. T., Kang D., Mathur R., Murphy B. N., Napelenok S. L., Nolte C. G., Pleim J. E., Pouliot G. A., Pye H. O. T., Ran L., Roselle S. J., Sarwar G., Schwede D. B., Sidi F. I., Spero T. L., Wong D. C. (2021). The Community Multiscale
Air Quality (CMAQ) model versions
5.3 and 5.3.1: system updates and evaluation. Geosci. Model Dev..

[48] Wang L., Wei Z., Wei W., Fu J. S., Meng C., Ma S. (2015). Source apportionment
of PM_2.5_ in top polluted cities in Hebei, China using the
CMAQ model. Atmos. Environ..

[49] Thongthammachart T., Araki S., Shimadera H., Eto S., Matsuo T., Kondo A. (2021). An integrated model combining random
forests and WRF/CMAQ model for
high accuracy spatiotemporal PM_2.5_ predictions in the Kansai
region of Japan. Atmos. Environ..

[50] Zhang Q., Xue D., Liu X., Gong X., Gao H. (2019). Process analysis of
PM_2.5_ pollution events in a coastal city of China using
CMAQ. Journal of Environmental Sciences.

[51] U.S. EPA Office of Research and Development . CMAQ. 5.3.2; U.S. EPA Office of Research and Development: Washington, D.C., 2020.

[52] McNamara, D. ; Stephens, G. ; Ruminski, M. ; Kasheta, T. The Hazard Mapping System (HMS)-NOAA multi-sensor fire and smoke detection program using environmental satellites. Proceedings of the 13th Conference on Satellite Meteorology and Oceanography; Norfolk, VA, Sept 20–23, 2004.

[53] Eidenshink J., Schwind B., Brewer K., Zhu Z.-L., Quayle B., Howard S. (2007). A project for monitoring trends in burn severity. Fire ecology.

[54] Walters, S. P. ; Schneider, N. J. ; Guthrie, J. D. Geospatial Multi-Agency Coordination (GeoMAC) Wildland Fire Perimeters, 2008; U.S. Geological Survey: Reston, VA, 2011; Data Series 612, pp 6, 10.3133/ds612.

[55] Meng X., Garay M. J., Diner D. J., Kalashnikova O. V., Xu J., Liu Y. (2018). Estimating PM_2.5_ speciation concentrations
using prototype 4.4 km-resolution MISR aerosol properties over Southern
California. Atmos. Environ..

[56] Childs M. L., Li J., Wen J., Heft-Neal S., Driscoll A., Wang S., Gould C. F., Qiu M., Burney J., Burke M. (2022). Daily Local-Level
Estimates of Ambient Wildfire Smoke PM_2.5_ for the Contiguous
US. Environ. Sci. Technol..

[57] Wei J., Li Z., Chen X., Li C., Sun Y., Wang J., Lyapustin A., Brasseur G. P., Jiang M., Sun L., Wang T., Jung C. H., Qiu B., Fang C., Liu X., Hao J., Wang Y., Zhan M., Song X., Liu Y. (2023). Separating
Daily 1 km PM_2.5_ Inorganic Chemical Composition
in China since 2000 via Deep Learning Integrating Ground, Satellite,
and Model Data. Environ. Sci. Technol..

[58] Lyapustin A., Wang Y., Korkin S., Huang D. (2018). MODIS Collection 6
MAIAC algorithm. Atmos. Meas. Technol..

[59] Chawla N. V., Bowyer K. W., Hall L. O., Kegelmeyer W. P. (2002). SMOTE:
synthetic minority over-sampling technique. Journal of artificial intelligence research.

[60] Geng G., Meng X., He K., Liu Y. (2020). Random forest models
for PM_2.5_ speciation concentrations using MISR fractional
AODs. Environmental Research Letters.

[61] Ploton P., Mortier F., Réjou-Méchain M., Barbier N., Picard N., Rossi V., Dormann C., Cornu G., Viennois G., Bayol N. (2020). Spatial
validation reveals poor predictive performance of large-scale ecological
mapping models. Nat. Commun..

[62] Hao H., Wang Y., Zhu Q., Zhang H., Rosenberg A., Schwartz J., Amini H., van Donkelaar A., Martin R., Liu P. (2023). National
cohort study
of long-term exposure to PM_2.5_ components and mortality
in Medicare American older adults. Environ.
Sci. Technol..

[63] U.S. Department of Health and Human Services (HHS) . HHS Standard Values for Regulatory Analysis, 2024; HHS: Washington, D.C., 2024.

[64] Judek, S. ; Stieb, D. ; Jovic, B. ; Edwards, B. Air Quality Benefits Assessment Tool (AQBAT) User Guide: Version 2; Health Canada: Ottawa, Ontario, Canada, 2012.

[65] Crouse D. L., Peters P. A., van Donkelaar A., Goldberg M. S., Villeneuve P. J., Brion O., Khan S., Atari D. O., Jerrett M., Pope C. A. (2012). Risk of nonaccidental
and cardiovascular mortality in relation to long-term exposure to
low concentrations of fine particulate matter: a Canadian national-level
cohort study. Environ. Health Perspect..

[66] Chestnut, L. ; De Civita, P. Economic Valuation of Mortality Risk Reduction; Government of Canada: Ottawa, Ontario, Canada, 2009; pp 1–69.

[67] Cummins K., Noble J., Varner J. M., Robertson K. M., Hiers J. K., Nowell H. K., Simonson E. (2023). Simonson, E. The Southeastern
U.S. Prescribed Fire Permit Database: Hot Spots and Hot Moments in
Prescribed Fire across the Southeastern U.S.A. Fire.

[68] Rogers H. M., Ditto J. C., Gentner D. R. (2020). Evidence for impacts on surface-level
air quality in the northeastern US from long-distance transport of
smoke from North American fires during the Long Island Sound Tropospheric
Ozone Study (LISTOS) 2018. Atmospheric Chemistry
and Physics.

[69] Wang Z., Huang X., Xue L., Ding K., Lou S., Zhu A., Ding A. (2024). Intensification of mid-latitude cyclone by aerosol-radiation
interaction increases transport of Canadian wildfire smoke to northeastern
US. Geophys. Res. Lett..

[70] Brown T., Leach S., Wachter B., Gardunio B. (2020). The Northern California
2018 extreme fire season. Bulletin of the American
Meteorological Society.

[71] Zhai J., Ning Z., Dahal R., Yang S. (2023). Wildfire Susceptibility
of Land Use and Topographic Features in the Western United States:
Implications for the Landscape Management. Forests.

[72] Williams A. P., Abatzoglou J. T., Gershunov A., Guzman-Morales J., Bishop D. A., Balch J. K., Lettenmaier D. P. (2019). Observed
impacts of anthropogenic climate change on wildfire in California. Earth’s Future.

[73] Jain P., Castellanos-Acuna D., Coogan S. C., Abatzoglou J. T., Flannigan M. D. (2022). Observed
increases in extreme fire weather driven by
atmospheric humidity and temperature. Nature
Climate Change.

[74] Halofsky J. E., Peterson D. L., Harvey B. J. (2020). Changing wildfire,
changing forests:
the effects of climate change on fire regimes and vegetation in the
Pacific Northwest, USA. Fire Ecology.

[75] Westerling A. L., Hidalgo H. G., Cayan D. R., Swetnam T. W. (2006). Warming and earlier
spring increase western US forest wildfire activity. science.

[76] Yu G., Feng Y., Wang J., Wright D. B. (2023). Performance of Fire
Danger Indices and Their Utility in Predicting Future Wildfire Danger
Over the Conterminous United States. Earth’s
Future.

[77] Di
Virgilio G., Evans J. P., Blake S. A., Armstrong M., Dowdy A. J., Sharples J., McRae R. (2019). Climate change increases
the potential for extreme wildfires. Geophys.
Res. Lett..

[78] Liu Y., Liu Y., Fu J., Yang C.-E., Dong X., Tian H., Tao B., Yang J., Wang Y., Zou Y., Ke Z. (2022). Projection
of future wildfire emissions in western USA under climate change:
contributions from changes in wildfire, fuel loading and fuel moisture. International Journal of Wildland Fire.

[79] Munoz-Alpizar R., Pavlovic R., Moran M. D., Chen J., Gravel S., Henderson S. B., Ménard S., Racine J., Duhamel A., Gilbert S., Beaulieu P.-A., Landry H., Davignon D., Cousineau S., Bouchet V. (2017). Multi-Year (2013–2016) PM_2.5_ Wildfire
Pollution Exposure over North America as Determined
from Operational Air Quality Forecasts. Atmosphere.

[80] Pavlovic R., Chen J., Anderson K., Moran M. D., Beaulieu P.-A., Davignon D., Cousineau S. (2016). The FireWork
air quality forecast
system with near-real-time biomass burning emissions: Recent developments
and evaluation of performance for the 2015 North American wildfire
season. J. Air Waste Manage. Assoc..

[81] American Lung Association . State of the Air 2020; American Lung Association: Chicago, IL, 2020.

[82] Burke M., Childs M. L., de la Cuesta B., Qiu M., Li J., Gould C. F., Heft-Neal S., Wara M. (2023). The contribution of
wildfire to PM_2.5_ trends in the USA. Nature.

[83] Hanes C. C., Wang X., Jain P., Parisien M.-A., Little J. M., Flannigan M. D. (2019). Fire-regime changes in Canada over
the last half century. Canadian Journal of Forest
Research.

[84] Coogan S. C., Robinne F.-N., Jain P., Flannigan M. D. (2019). Scientists’
warning on wildfire—A Canadian perspective. Canadian Journal of Forest Research.

[85] Wang Z., Wang Z., Zou Z., Chen X., Wu H., Wang W., Su H., Li F., Xu W., Liu Z. (2024). Severe Global Environmental Issues
Caused by Canada’s Record-Breaking
Wildfires in 2023. Adv. Atmos. Sci..

[86] Carter T. S., Heald C. L., Jimenez J. L., Campuzano-Jost P., Kondo Y., Moteki N., Schwarz J. P., Wiedinmyer C., Darmenov A. S., da Silva A. M., Kaiser J. W. (2020). How emissions
uncertainty
influences the distribution and radiative impacts of smoke from fires
in North America. Atmospheric Chemistry and
Physics Discussions.

[87] Tian X., Zhao F., Shu L., Wang M. (2013). Distribution characteristics
and the influence factors of forest fires in China. Forest Ecology and Management.

[88] Valkó O., Deák B. (2021). Increasing
the potential of prescribed burning for
the biodiversity conservation of European grasslands. Current Opinion in Environmental Science & Health.

[89] Weir, J. R. ; Scasta, J. D. Global Application of Prescribed Fire; CSIRO Publishing: Clayton, Australia, 2022.

[90] Kolden C. A. (2019). We’re
Not Doing Enough Prescribed Fire in the Western United States to Mitigate
Wildfire Risk. Fire.

[91] Engebretson J. M., Hall T. E., Blades J. J., Olsen C. S., Toman E., Frederick S. S. (2016). Characterizing
public tolerance of smoke from wildland
fires in communities across the United States. Journal of Forestry.

[92] U.S. Department of the Interior . Budget Justifications and Performance Information Fiscal Year 2024: Wildland Fire Management; U.S. Department of the Interior: Washington, D.C., 2024; https://www.doi.gov/media/document/fy2024-wfm-greenbook-508-pdf.

[93] Wiedinmyer C., Hurteau M. D. (2010). Prescribed fire
as a means of reducing forest carbon
emissions in the western United States. Environ.
Sci. Technol..

[94] Arkle R. S., Pilliod D. S., Welty J. L. (2012). Pattern and process of prescribed
fires influence effectiveness at reducing wildfire severity in dry
coniferous forests. Forest Ecology and Management.

[95] Tolhurst K. G., McCarthy G. (2016). Effect of prescribed
burning on wildfire severity:
a landscape-scale case study from the 2003 fires in Victoria. Australian Forestry.

[96] Williamson G. J., Bowman D. M. J. S., Price O. F., Henderson S. B., Johnston F. H. (2016). A transdisciplinary
approach to understanding the health
effects of wildfire and prescribed fire smoke regimes. Environmental Research Letters.

[97] Macias
Fauria M., Johnson E. (2008). Climate and wildfires in the North
American boreal forest. Philosophical Transactions
of the Royal Society B: Biological Sciences.

