# Supplementary material for: Fire Smoke Elevated the Carbonaceous PM2.5 Concentration and Mortality Burden in the Contiguous U.S. and Southern Canada
Source: Environ Sci Technol. 2025 Jun 12;59(24):12196–210. doi: 10.1021/acs.est.5c01641 (PMC12199464; doi:10.1021/acs.est.5c01641)
Supplement: Supplementary file 1 [file es5c01641_si_001.pdf]

Supplemental Information for

Fire Smoke Elevated the Carbonaceous $PM_{2.5}$ Concentration and Mortality Burden in the Contiguous U.S. and Southern Canada

Zhihao Jin[1], Gonzalo A. Ferrada[2,4], Danlu Zhang[3], Noah Scovronick[1], Joshua S. Fu[2], Kai Chen[5,6], Yang Liu[1,]*

[1]Gangarosa Department of Environmental Health, Rollins School of Public Health, Emory University, Atlanta, GA 30322, USA
[2]Deparent of Civil and Environmental Engineering, University of Tennessee, Knoxville, TN 37996, USA
[3]Deparent of Biostatistics, Rollins School of Public Health, Emory University, Atlanta, GA 30322, USA
[4]Global Systems Laboratory, NOAA Earth System Research Laboratories, Boulder, CO 80305, USA
[5]Department of Environmental Health Sciences, Yale School of Public Health, New Haven, CT 06510, USA
[6]Yale Center on Climate Change and Health, Yale School of Public Health, New Haven, CT 06510, USA

Supplemental Information Summary

Supplemental information includes 32 pages, 17 tables, and 15 figures.

Supplemental Materials and Methods

S1. Auxiliary predictor pre-processing

We matched 1×1 km^2 grid cells with predictors of the same spatial resolution, including the Multi-Angle Implementation of Atmospheric Correction (MAIAC) aerosol optical depth (AOD), the Moderate Resolution Imaging Spectroradiometer (MODIS) normalized difference vegetation index (NDVI), enhanced vegetation index (EVI) and cloud fraction, and population density data obtained from the Landscan Program at Oak Ridge National Laboratory (ORNL), and human footprint data from Mu et al.'s work.[1] Due to missing data in the MAIAC AOD, we applied a two-step gap-filling approach to achieve full coverage of the daily MAIAC AOD dataset.

For predictors with coarser spatial resolution (i.e., > 1×1 km^2), including the CMAQ simulations, the Copernicus Atmosphere Monitoring Service (CAMS) AOD and biogenic emissions, the Multi-angel Imaging Spectro Radiometer (MISR) AOD, and the meteorological variables from the European Centre for Medium-Range Weather Forecasts Reanalysis v5 (ERA-5) and the North American Regional Reanalysis (NARR). We interpolated them into the 1×1 km^2 grid cells using inverse distance weighting (IDW).

For higher spatial resolution predictors (i.e., < 1×1 km^2), such as land cover data from the National Land Cover Database, road network from the Global Roads Inventory Project, and elevation data from the U.S. Geological Survey's (USGS) National Geospatial Program, we aggregated the data to the 1×1 km grid cells. Specifically, we calculated the percentages of land cover types, the average elevations, and the total road length of different road types within each grid cell. In addition, we computed daily total smoke plumes duration and weighed average plume density for each grid cell based on the fire smoke polygons produced by the NOAA Hazard Mapping System (HMS). To address missing data in the HMS smoke density and duration, we implemented a gap-filling approach to achieve full coverage of the daily HMS dataset.

Since smoke air pollutants exhibit significant spatiotemporal heterogeneity, incorporating this variability into models was critical for improving predictive accuracy. We included optimized spatiotemporal information by employing weighted effects referencing polar coordinates.[2] The unequal spatial autocorrelation and differences across grid cells were represented in Euclidean space using three spherical coordinates [*S1, S2, S3*]. The temporal characteristics of the grid cells were captured using three helix-shaped trigonometric vectors [*T1, T2, T3*], accounting for both seasonal cycles and daily variations of concentrations.

$$S1_s, S2_s, S3_s = \left[\cos\left(2\pi\frac{Lon_s}{360}\right)\cos\left(2\pi\frac{Lat_s}{180}\right), \cos\left(2\pi\frac{Lon_s}{360}\right)\sin\left(2\pi\frac{Lat_s}{180}\right), \sin\left(2\pi\frac{Lon_s}{360}\right)\right] \quad [1]$$

$$T1_t, T2_t, T3_t = \left[\frac{DOY_t}{N}, \cos\left(2\pi\frac{DOY_t}{N}\right), \sin(2\pi)\right] \quad [2]$$

Where Lon_s and Lat_s are the longitude and latitude of the grid cell s, respectively, and DOY_t and N represent the day of the year of the date t and the total number of days in the year, respectively.

S1.1 MAIAC AOD Gap-filling approach

To address the missing data in MAIAC AOD, we implemented a two-step approach to enhance the spatial coverage of the satellite AOD data. First, we employed linear regression models to fit the relationship between daily Terra and Aqua AOD measurements, enabling the predictions of missing Terra AOD where only Aqua AOD was available, and vice versa. The daily averaged AOD in each grid was calculated as the means of both Terra and Aqua satellite measurements. Second, we developed a random forest (RF) model to predict daily AOD in grid cells where both Terra and Aqua AOD were missing, using the following equation:

$$AOD_{(s,t)} = f(cloud\ fraction_{(s,t)}, air\ temperature_{(s,t)}, specific\ humidity_{(s,t)},$$
$$precipitation_{(s,t-1)}, elevation_{(s)}, auxillary\ AOD_{(s,t)}) \quad [3]$$

Where s and t are the grid cell location and date of observation, respectively. $cloud\ fraction$, $air\ temperature$, $specific\ humidity$, and $precipitation$ are extracted from MODIS and ERA-5 datasets. To improve the gap-filling accuracy, we incorporated $auxillary\ AOD$ data obtained from the

Copernicus Atmosphere Monitoring (CAMS), which provides AOD measurements at five wavelengths every three hours. For the year 2002, when CAMS data was unavailable, we used Modern-Era Retrospective analysis (MERRA-2) AOD as a substitute. Because both CAMS and MERRA-2 AOD products have a coarser spatial resolution than 1×1 km^2, we interpolated these AOD measurements to the 1×1 km^2 grid cells using IDW before incorporating into the RF models. MAIAC AOD values at 470 and 550 nm were modeled separately on each day.

To balance computing efficiency with prediction accuracy, we simplified our RF gap-filling models by predefining the number of trees (*num.tree*) and the number of variables randomly sampled as candidates at each split (*mtry*). Additionally, we limited the size of the training dataset to a maximum of 1,000,000 observations per day. If the total number of available observations exceeded this limit, 1,000,000 observations were randomly selected for training.

S1.2 HMS Smoke Plume Gap-filling Approach

The HMS smoke plume products provide daily information on the location, density, and duration of fire smoke from 2005-2019. For periods without HMS data (i.e., prior to 2005), fire scenario classification relied solely on the smoke $PM_{2.5}$ ratio. To interpolate the daily density and duration of smoke for the Smoke-on grids during these periods, we trained the Extremely Randomized Trees (EXT) models for each day, as EXT is more efficient than RF when handling large datasets. Because the HMS smoke plume provides fewer observations in non-wildfire seasons, which may limit the gap-filling performance. We therefore created the training datasets using data from five years (2006-2010) with three consecutive days: the same day of year, the previous day of year, and the next day of year, as following equation:

$$Smoke\ density\ or\ duration_{s,t} = f(Fireon\ PM_{2.5}\ Mass_{s,T}, Fireoff\ PM_{2.5}\ Mass_{s,T}, cloud\ fraction_{(s,T)}, planet\ boundary\ layer\ height_{(s,T)}, NDVI_{(s,T)}, Uwind_{(s,T)}, Vwind_{(s,T)}, S_{1_s}, S_{2_s}, S_{3_s},) \quad [4]$$

Where s and t are the grid cell location and date of a prediction of smoke density or duration, respectively. T is the three consecutive days, including t-1, t, and t+1. $Fireon\ PM_{2.5}\ Mass$ and

$Fireoff\ PM_{2.5}\ Mass$ were obtained from the CMAQ simulations; $cloud\ fraction$, air temperature, $planet\ boundary\ layer\ height$, $NDVI$, $Uwind$ and $Vwind$ are extracted from MODIS and ERA-5 datasets. , $S1_s$, $S2_s$, $S3_s$ are the three spherical coordinates. To improve the efficiency of the EXT models, we used consistent hyper-parameters and limited the size of the training dataset to no more than 5,000,000 observations per day. Here we used a higher number of observations than the gap filling for MAIAC AOD, because more days of observations were included.

S2. Modeling framework

S2.1 SMOTE

SMOTE is an oversampling technique which generates synthetic samples for the underrepresented class, which accounted for only a small proportion of ground-based measurements but played an important role in improving predicting at high ranges. It operates by generating new instances in the feature space from the nearest neighboring grid cells (five nearest neighbors in our study) in the training data set.[3] For $PM_{2.5}$ OC and EC, we set the underrepresented class as concentrations between the 99th and 99.9^{th} percentiles (OC: 7.36 – 18.42 μg/m^3; EC: 2.03 – 4.22 μg/m^3) and the measurements above the 99.9^{th} percentile (OC: > 18.42 μg/m^3; EC: > 4.22 μg/m^3), which were oversampled once and twice, respectively. The oversampling process added 1.1% of the initial dataset, without skewing the overall distribution of $PM_{2.5}$ observations. After SMOTE pre-processing, the datasets were classified into Smoke-on and Smoke-off scenarios and subsequently fed into the RASL framework.

S2.2 Level 1

In level 1, we adopted four machine learning algorithms as base learners, selected based on their balance of accuracy and computational efficiency. In the realm of machine learning, ensemble methods such as Extreme Gradient Boosting (XGB), Light Gradient Boosting Machine (LGB), Random Forest (RF), and Extremely Randomized Trees (EXT) are widely recognized for their robust predictive capabilities.[4-7] These methods are widely used in air pollution modeling.[8-10] The hyper-parameters of each

base learner were optimized using random search with repeated 3-fold cross-validation. The number of search iterations varied across models, ranging from 50 for EXT to 500 for XGB, depending on the complexity of the hyperparameters. After tuning the hyperparameters, we randomly split each training dataset into 10 folds.

S2.3 Level 2

To leverage the strengths of the base learners and enhance prediction accuracy, we applied meta-learners to integrate the predictions from the base learners. Similar to level 1, we used XGB, LGB, RF, and EXT as candidate meta-learners. A grid search strategy was employed to tune the meta-learners, and the tuned meta-learner with the best performance was selected as the final meta-learner. The 10-fold cross-validated predictions from the base learners were used to tune and fit the meta-learners. The integrated prediction from meta-learner is represented by the following equation:

$$Pred_{Meta\ Learner_{(s,t)}} = f\left(Pred_{XGB_{(s,t)}}, Pred_{LGB_{(s,t)}}, Pred_{RF_{(s,t)}}, Pred_{EXT_{(s,t)}}\right) \quad [5]$$

where: $Pred_{XGB_{(s,t)}}$, $Pred_{LGB_{(s,t)}}$, $Pred_{RF_{(s,t)}}$ and $Pred_{EXT_{(s,t)}}$ are the cross-validated predictions from XGB, LGB, RF and EXT, respectively; $Pred_{Meta\ Learner_{(s,t)}}$ is the integrated prediction from the meta-learner.

S2.4 Level 3

To further refine the predictions, we employed generalized additive models (GAMs) to analyze the spatiotemporal residuals of the Level-2 meta-learner predictions at the monthly level. GAMs are a class of statistical models that extend generalized linear models by allowing non-linear relationships between the predictors and the response variable, which are modeled using smooth functions, such as splines.[11] Compared to a simple global correction such as adding a correction term across the entire domain, GAMs can flexibly model smooth nonlinear variation across space.

We adjusted at the monthly scale because the speciation monitors generally collected one 24-hour sample every 3 days, it is not practical to adjust residual for every day.[12, 13] By averaging each grid cell's daily predictions into monthly means and then correcting those means, we also reduced day-to-day noise

and captured monthly biases more reliably. In addition, the daily "Smoke-on" classification is not suitable at the monthly level, because most grid cells experience a mixture of Smoke-on and Smoke-off days within any given month. Averaging only the "Smoke-on" observations or predictions would only reflect pollution level exclusively on smoke impacted days, rather than the month's overall smoke impacts. We instead used two monthly concentration metrics: "Background" and "Total". "Background" concentration is calculated by assuming every day as Smoke-off scenario, and "Total" concentration is defined as the mean of combined Smoke-off and Smoke-on days (**Equation 6**). The monthly smoke contribution was then derived as the difference between Total and Background concentrations.

$$Background\ PM_{2.5_{(s,m)}} = \frac{\sum_t PM_{2.5_{(s,m,t)}}^{Smoke-off}}{Number\ of\ Days\ in\ Month_m}$$

$$Total\ PM_{2.5_{(s,m)}} = \frac{\sum_t (PM_{2.5_{(s,m,t)}}^{Smoke-off}\ or\ PM_{2.5_{(s,m,t)}}^{Smoke-on})}{Number\ of\ Days\ in\ Month_m} \quad [6]$$

where: $Background$ and $Total\ PM_{2.5_{(s,m)}}$ are the monthly Background and Total concentration at location s in month m. $PM_{2.5_{(s,m,t)}}^{Smoke-off}$ and $PM_{2.5_{(s,m,t)}}^{Smoke-on}$ are the daily $PM_{2.5}$ concentration at location s on the month-day (m, t).

To fit the GAMs, we first computed the monthly residuals (i.e., Background or Total concentration) by calculating each ground monitor's monthly observation and subtracting the corresponding meta-learner's monthly predictions for the same location and month (**Equation 7**).

$$Residual_{(s,m)}^{Background\ or\ Total} = Observation_{(s,m)}^{Background\ or\ Total} - Prediction_{(s,m)}^{Background\ or\ Total} \quad [7]$$

where: $Observation_{(s,m)}$ and $Prediction_{(s,m)}$ are the monthly observation and prediction at location s in month m. $Residual_{(s,m)}$ is the monthly residual error.

We then regressed these residuals against three spherical coordinates of the monitor locations to fit separate GAM for every month and both Background and Total concentrations. The fitted GAMs were projected (i.e., added back) to the monthly meta-learner predictions, yielding bias-corrected concentrations for all 1 × 1 km^2 grid cells. This monthly, GAM-based adjustment accounted for spatial

and temporal dependencies that might not have been captured after Level-1 and Level-2 modeling steps, leading to more accurate and robust long-term predictions. The residual modeling is expressed as follows:

$$Residual_{(s,m)}^{Background\ or\ Total} = \mathrm{f}_{GAM}\ [\ smooth_1(S_{1_s}), smooth_2(S_{2_s}), smooth_3(S_{3_s})\ ] \qquad [8]$$

$$Adjusted\ Prediction_{(s,m)}^{Background\ or\ Total} = Prediction_{(s,m)}^{Background\ or\ Total} + GAM\ Adjustment_{(s,m)}^{Background\ or\ Total} \qquad [9]$$

where: $smooth_i$ represents the smooth functions with i = 1, 2, 3, each modeled with 20 splines. S_1, S_2, and S_3 are the three spherical coordinates. Python package "pygam" was used for GAMs.

S3. Model Performance Evaluation

In the first stage, the dataset was divided into groups according to the CV type, with one group used as the testing dataset and the rest as the training dataset for building base learners. This process was repeated across all groups, and fitted results from testing datasets were combined. In the second stage, the combined predictions of the base learners were used to build a meta-learner model. This was similarly divided for training and testing, and repeated iterations produced the final meta-learner predictions. In the third stage, GAM models were fitted for each month using the meta-learner residuals to adjust the predictions on a month-by-month basis, specifically to correct systematic errors in residuals. Because the monthly GAM required averaging daily observations at each monitor, which can not be embedded in the CV loops used for the daily models at stage 1 and 2, we focused instead on improving robustness and correcting residual errors from stage 2. The performance of GAM models was evaluated based on cross-validated monthly Background and Total concentrations.

S4. Sensitivity Analyses for CONUS Mortality Burden

The annual baseline mortality data for CONUS counties from 2003-2020 was obtained from the National Center for Health Statistics and matched to our modeled smoke OC and EC concentrations after aggregating the grids to county means.[14] The mortality burden was then estimated in three steps.

First, we adopted concentration–response results from the epidemiological study by Hao et al., which investigated the impact of ambient $PM_{2.5}$ speciation on mortality based the Medicare American older adults. We used the hazard ratios (HRs) reported by Hao et al., including black carbon (BC) (HR = 1.059 [95% CI: 1.057 –1.060]) and organic matter (OM) (HR = 1.017 [95% CI: 1.017–1.017]). BC was treated as a proxy for smoke EC, while OM was used as a proxy for smoke OC after converting from OM mass to OC mass based on the widely adopted ratio OM:OC = 1.8:1.[15, 16] We therefore rescaled the HR and coefficient of OM to OC by:

$$HR_{OC} = {HR_{OM}}^{1.8} = 1.031\ per\ 1\ ug/m^3$$

$$\beta_{OC} = 3.03 \times 10^{-2}$$

$$\beta_{EC} = \ln(1.059) = 5.73 \times 10^{-2}$$

Second, we computed the county-specific relative risk (RR) for OC or EC as:

$$RR_{c,t,k} = \exp\left(\beta_k C_{c,t,k}\right)$$

Where k is the $PM_{2.5}$ OC or EC, β_k is the effect coefficient of OC or EC, $C_{c,t,k}$ is the annual mean OC or EC for county c in year t. The corresponding attributable fraction (AF) of deaths is calculated by:

$$AF_{c,t,k} = 1 - RR_{c,t,k}^{-1}$$

Third, we calculated the attributable deaths, D, as:

$$D_{c,t,k} = Baseline\ Mortality_{c,t} \times AF_{c,t,k}$$

To isolate the smoke contribution, we performed the calculation twice — once with total (background + smoke) component concentrations and then with background-only concentrations, and compute the difference, i.e.,

$$D_{c,t,k}^{smoke} = D_{c,t,k}^{total} - D_{c,t,k}^{background}$$

Summing $D_{c,t,k}^{smoke}$ over all counties yielded the annual deaths attributable to smoke-derived EC and OC in CONUS.

Supplemental Tables

Table S1. The numbers of ground-based stations and observations for training datasets by year and smoke scenario.

Year	# OC Observations				# EC Observations			
	Stations	Total	Smoke-on (%)	Smoke-off	Stations	Total	Smoke-on (%)	Smoke-off
2002	186	20,406	4,460 (21.9)	15,946	186	20,413	4,467 (21.9)	15,946
2003	200	22,324	7,710 (34.5)	14,614	200	22,280	7,700 (34.6)	14,580
2004	217	24,345	6,887 (28.3)	17,458	217	24,327	6,882 (28.3)	17,445
2005	220	24,662	7,478 (30.3)	17,184	221	24,769	7,523 (30.4)	17,246
2006	217	24,374	8,682 (35.6)	15,692	217	24,361	8,675 (35.6)	15,686
2007	216	24,104	9,157 (38.0)	14,947	216	24,102	9,154 (38.0)	14,948
2008	218	24,070	9,018 (37.5)	15,052	218	24,083	9,032 (37.5)	15,051
2009	214	23,672	7,385 (31.2)	16,287	214	23,676	7,388 (31.2)	16,288
2010	209	22,640	6,559 (29.0)	16,081	209	22,642	6,561 (29.0)	16,081
2011	204	21,698	8,606 (39.7)	13,092	204	21,704	8,611 (39.7)	13,093
2012	202	22,667	9,430 (41.6)	13,237	202	22,676	9,439 (41.6)	13,237
2013	202	22,073	8,270 (37.5)	13,803	202	22,077	8,274 (37.5)	13,803
2014	202	22,279	7,026 (31.5)	15,253	202	22,278	7,025 (31.5)	15,253
2015	317	22,661	8,747 (38.6)	13,914	317	22,670	8,755 (38.6)	13,915
2016	317	31,275	11,537 (36.9)	19,738	317	31,280	11,540 (36.9)	19,740
2017	319	31,666	16,048 (50.7)	15,618	319	31,690	16,073 (50.7)	15,617
2018	319	32,126	16,066 (50.0)	16,060	319	32,128	16,068 (50.0)	16,060
2019	320	31,586	13,126 (41.6)	18,460	320	31,586	13,126 (41.6)	18,460
Total	400	448,628	166,192 (37.0)	282,436	401	448,742	166,293 (37.0)	282,449
SMOTE		453,564	170,201 (37.5)	283,363		453,679	169,052 (37.3)	284,627

Note: New stations were built at the end of 2015, resulting in more stations in 2015 but not more observations.

Table S2. Annual mean concentrations of ground-based observations for training datasets by year and smoke scenarios (Unit: μg/m^3).

**Year**	**OC Concentrations (μg/m^3)**			**EC Concentrations (μg/m^3)**		
	Total	Smoke-on	Smoke-off	Total	Smoke-on	Smoke-off
2002	1.395	2.372	1.122	0.282	0.380	0.255
2003	1.441	1.912	1.192	0.294	0.321	0.279
2004	1.404	1.746	1.269	0.295	0.322	0.284
2005	1.384	1.652	1.267	0.380	0.409	0.367
2006	1.418	1.790	1.212	0.359	0.423	0.323
2007	1.432	1.927	1.129	0.323	0.398	0.277
2008	1.261	1.686	1.007	0.270	0.320	0.241
2009	1.147	1.437	1.015	0.250	0.275	0.238
2010	1.026	1.434	0.860	0.225	0.287	0.200
2011	1.057	1.392	0.837	0.214	0.253	0.188
2012	1.119	1.491	0.854	0.217	0.251	0.192
2013	0.953	1.268	0.764	0.189	0.223	0.168
2014	0.923	1.277	0.759	0.182	0.211	0.168
2015	1.160	1.629	0.865	0.198	0.238	0.173
2016	1.290	1.659	1.075	0.290	0.329	0.268
2017	1.616	2.075	1.145	0.340	0.386	0.291
2018	1.545	1.998	1.091	0.363	0.412	0.313
2019	1.259	1.561	1.045	0.370	0.416	0.338
Average	1.284	1.702	1.037	0.286	0.335	0.258

Note: The results were summarized before the SMOTE process.

Table S3. Summary statistics of ground-based observations for training datasets from 2002 to 2019 (Unit: μg/m^3).

**$PM_{2.5}$ speciation**	**Min**	**1st Quantile**	**Median**	**Mean**	**3rd Quantile**	**Max**
OC	< 0.0001	0.379	0.831	1.284	1.588	39.340
EC	< 0.0001	0.074	0.152	0.286	0.338	7.392

Table S4. Main configurations of the CMAQ simulations.

Parameter	Value	Reference(s)
Version	5.3.2.1	Appel et al.[17]
Domain coverage	Continental US, southern Canada and northern Mexico	
Horizontal resolution	12 km	
Vertical layers	35	
Gas-phase chemistry	Carbon bond v6.3 (CB6R3)	Pye et al.[18]
Aerosols chemistry	AE7	Xu et al.[19]
Aqueous and heterogeneous chemistry	Yes	Pye et al.[18, 20]
Ammonia bi-directional flux	Yes	Appel et al.[17]
Lightning NOx	Yes	Appel et al.[17]
Online Biogenic emissions	Yes	Xu et al.[19]
Ocean halogen chemistry	Yes	Sarwar et al.[21]

Table S5. Correlations between ground-based observations of $PM_{2.5}$ OC and EC for training datasets and corresponding CMAQ simulations under two smoke scenarios.

Ground-based Observations	Correlation with Corresponding CMAQ Simulations
OC: Smoke-on	0.32
OC: Smoke-off	0.62
EC: Smoke-on	0.55
EC: Smoke-off	0.74

Note: Extreme OC and EC values (> 99.98th percentile) were removed.

Table S6. Association between 12-month moving average concentration of smoke $PM_{2.5}$ and monthly mortality rate, with estimated annual mortality rate and 95% confidence intervals.[22]

12-Month average of smoke $PM_{2.5}$ concentration (μg/m³)	Change in monthly mortality rate (per 100,000 people)	Change in annual mortality rate (per 100,000 people)
0.1-0.2	0.16 (0.06, 0.26)	1.92 (0.72, 3.12)
0.2-0.3	0.40 (0.26, 0.54)	4.80 (3.12, 6.48)
0.3-0.4	0.35 (0.21, 0.49)	4.20 (2.52, 5.88)
0.4-0.5	0.34 (0.19, 0.50)	4.08 (2.20, 5.96)
0.5-0.7	0.49 (0.33, 0.65)	5.88 (3.60, 8.16)
0.7-1.0	0.63 (0.44, 0.83)	7.56 (5.28, 9.84)
1.0-5.0	0.36 (0.11, 0.61)	4.32 (1.32, 7.32)
5.0 +	2.11 (1.24, 2.99)	25.32 (14.82, 35.82)

Table S7. 10-fold random cross-validation (CV) results for base learners.

$PM_{2.5}$ Speciation	Smoke Scenario	Model	Random CV								
			Daily			Monthly			Yearly		
			RMSE	R^2	Slope	RMSE	R^2	Slope	RMSE	R^2	Slope
OC	Smoke-off	XGB	0.562	0.773	1.023	0.333	0.884	1.038	0.131	0.976	1.021
		LGB	0.566	0.769	1.017	0.345	0.875	1.029	0.135	0.974	1.017
		RF	0.591	0.749	1.027	0.372	0.855	1.040	0.177	0.956	1.026
		EXT	0.589	0.751	1.049	0.370	0.858	1.054	0.168	0.961	1.036
	Smoke-on	XGB	1.210	0.665	1.069	0.657	0.793	1.083	0.348	0.909	1.120
		LGB	1.241	0.645	1.041	0.675	0.780	1.066	0.367	0.895	1.105
		RF	1.320	0.599	1.052	0.754	0.727	1.080	0.448	0.846	1.145
		EXT	1.261	0.638	1.092	0.712	0.759	1.094	0.389	0.884	1.125
EC	Smoke-off	XGB	0.176	0.791	1.034	0.105	0.893	1.054	0.055	0.962	1.023
		LGB	0.172	0.800	1.019	0.099	0.905	1.040	0.047	0.972	1.011
		RF	0.183	0.773	1.026	0.112	0.879	1.060	0.062	0.952	1.024
		EXT	0.177	0.788	1.039	0.105	0.893	1.059	0.053	0.964	1.022
	Smoke-on	XGB	0.252	0.685	1.049	0.178	0.793	1.067	0.100	0.917	1.041
		LGB	0.243	0.706	1.016	0.162	0.826	1.032	0.073	0.956	1.019
		RF	0.260	0.665	1.037	0.187	0.770	1.051	0.109	0.901	1.027
		EXT	0.251	0.690	1.055	0.177	0.795	1.058	0.097	0.922	1.027

Note: RMSE represents root-mean-square error.

Table S8. 10-fold clustered spatial CV results for base learners.

$PM_{2.5}$ Speciation	Smoke Scenario	Model	10-fold Clustered Spatial CV								
			Daily			Monthly			Yearly		
			RMSE	R^2	Slope	RMSE	R^2	Slope	RMSE	R^2	Slope
OC	Smoke-off	XGB	0.809	0.540	1.169	0.631	0.594	1.191	0.488	0.680	1.193
		LGB	0.787	0.559	1.096	0.609	0.615	1.122	0.471	0.695	1.128
		RF	0.815	0.523	1.036	0.626	0.591	1.087	0.468	0.697	1.110
		EXT	0.792	0.556	1.131	0.612	0.616	1.156	0.462	0.710	1.156
	Smoke-on	XGB	1.453	0.517	1.092	0.916	0.599	1.111	0.672	0.644	1.227
		LGB	1.439	0.523	1.034	0.911	0.597	1.036	0.678	0.623	1.125
		RF	1.465	0.505	1.027	0.929	0.583	1.051	0.682	0.624	1.171
		EXT	1.466	0.507	1.063	0.932	0.586	1.055	0.675	0.633	1.139
EC	Smoke-off	XGB	0.249	0.573	1.036	0.186	0.644	1.067	0.144	0.727	1.032
		LGB	0.246	0.584	0.990	0.183	0.653	1.031	0.143	0.731	0.998
		RF	0.263	0.535	0.880	0.196	0.606	0.927	0.154	0.694	0.907
		EXT	0.254	0.556	0.991	0.192	0.619	1.005	0.150	0.705	0.968
	Smoke-on	XGB	0.307	0.550	1.024	0.248	0.603	1.003	0.192	0.693	0.954
		LGB	0.307	0.549	0.968	0.247	0.608	0.961	0.193	0.695	0.918
		RF	0.318	0.518	0.938	0.257	0.576	0.920	0.204	0.667	0.872
		EXT	0.310	0.541	1.028	0.252	0.594	0.972	0.199	0.679	0.910

Table S9. Leave-one-year-out temporal CV results for base learners.

$PM_{2.5}$ Speciation	Smoke Scenario	Model	Leave-one-year-out Temporal CV								
			Daily			Monthly			Yearly		
			RMSE	R^2	Slope	RMSE	R^2	Slope	RMSE	R^2	Slope
**OC**	Smoke-off	XGB	0.622	0.725	1.071	0.413	0.824	1.078	0.235	0.925	1.063
		LGB	0.611	0.732	1.034	0.404	0.830	1.047	0.224	0.930	1.037
		RF	0.637	0.708	1.029	0.424	0.812	1.046	0.246	0.915	1.033
		EXT	0.628	0.718	1.057	0.418	0.819	1.062	0.235	0.924	1.047
	Smoke-on	XGB	1.381	0.563	1.085	0.791	0.701	1.115	0.487	0.822	1.182
		LGB	1.366	0.569	1.029	0.775	0.708	1.058	0.475	0.820	1.111
		RF	1.399	0.549	1.032	0.812	0.681	1.068	0.504	0.800	1.148
		EXT	1.393	0.554	1.069	0.801	0.691	1.077	0.482	0.815	1.123
**EC**	Smoke-off	XGB	0.191	0.752	1.025	0.127	0.845	1.053	0.082	0.914	1.013
		LGB	0.189	0.758	1.007	0.124	0.850	1.038	0.081	0.918	0.999
		RF	0.197	0.737	1.001	0.130	0.835	1.043	0.085	0.908	1.003
		EXT	0.192	0.751	1.026	0.127	0.844	1.050	0.080	0.919	1.006
	Smoke-on	XGB	0.270	0.638	1.043	0.198	0.741	1.055	0.127	0.863	1.022
		LGB	0.268	0.644	1.005	0.193	0.752	1.014	0.121	0.876	0.991
		RF	0.274	0.626	1.026	0.204	0.725	1.036	0.131	0.856	1.011
		EXT	0.271	0.637	1.045	0.198	0.740	1.042	0.126	0.865	1.006

Table S10. 10-fold random CV results for super learners.

$PM_{2.5}$ Speciation	Smoke Scenario	Model	10-fold Random CV								
			Daily			Monthly			Yearly		
			RMSE	R^2	Slope	RMSE	R^2	Slope	RMSE	R^2	Slope
**OC**	Smoke-off	XGB	0.564	0.771	1.004	0.340	0.878	1.015	0.135	0.974	0.999
		LGB	0.564	0.771	0.996	0.339	0.879	1.008	0.135	0.974	0.993
		RF	0.562	0.772	0.994	0.336	0.881	1.006	0.135	0.974	0.991
		EXT	0.560	0.774	0.999	0.338	0.880	1.008	0.135	0.974	0.992
	Smoke-on	XGB	1.225	0.653	0.998	0.651	0.793	1.026	0.327	0.913	1.048
		LGB	1.223	0.654	0.999	0.648	0.795	1.025	0.322	0.915	1.047
		RF	1.208	0.663	0.985	0.642	0.799	1.017	0.311	0.921	1.046
		EXT	1.202	0.666	1.003	0.645	0.797	1.029	0.327	0.913	1.057
**EC**	Smoke-off	XGB	0.174	0.794	0.997	0.100	0.903	1.024	0.048	0.971	0.994
		LGB	0.174	0.794	0.996	0.099	0.904	1.025	0.046	0.973	0.996
		RF	0.172	0.799	0.995	0.097	0.908	1.019	0.045	0.974	0.991
		EXT	0.171	0.801	1.002	0.098	0.906	1.023	0.046	0.973	0.994
	Smoke-on	XGB	0.243	0.707	1.010	0.164	0.822	1.019	0.077	0.950	0.997
		LGB	0.242	0.708	1.002	0.163	0.824	1.009	0.075	0.952	0.988
		RF	0.242	0.708	0.993	0.161	0.828	1.007	0.072	0.956	0.993
		EXT	0.241	0.712	1.004	0.161	0.828	1.016	0.073	0.955	0.998

Table S11. 10-fold clustered spatial CV results for super learners.

$PM_{2.5}$ Speciation	Smoke Scenario	Model	10-fold Clustered Spatial CV								
			Daily			Monthly			Yearly		
			RMSE	R^2	Slope	RMSE	R^2	Slope	RMSE	R^2	Slope
OC	Smoke-off	XGB	0.785	0.562	1.101	0.607	0.618	1.124	0.468	0.699	1.125
		LGB	0.785	0.561	1.090	0.606	0.618	1.114	0.467	0.698	1.115
		RF	0.789	0.554	1.070	0.608	0.615	1.100	0.467	0.697	1.105
		EXT	0.784	0.562	1.096	0.607	0.618	1.118	0.467	0.699	1.119
	Smoke-on	XGB	1.452	0.514	0.966	0.909	0.600	0.993	0.659	0.642	1.084
		LGB	1.452	0.515	0.964	0.909	0.600	0.991	0.660	0.641	1.081
		RF	1.456	0.512	0.971	0.913	0.597	0.993	0.661	0.640	1.085
		EXT	1.443	0.520	0.988	0.906	0.602	1.008	0.660	0.642	1.097
EC	Smoke-off	XGB	0.246	0.583	0.993	0.184	0.651	1.024	0.143	0.730	0.986
		LGB	0.246	0.582	0.990	0.184	0.650	1.022	0.144	0.728	0.986
		RF	0.247	0.580	0.981	0.184	0.650	1.017	0.144	0.729	0.981
		EXT	0.246	0.583	0.984	0.184	0.651	1.015	0.143	0.729	0.980
	Smoke-on	XGB	0.305	0.555	0.980	0.246	0.611	0.963	0.193	0.695	0.911
		LGB	0.306	0.554	0.974	0.246	0.611	0.958	0.193	0.696	0.907
		RF	0.308	0.548	0.969	0.246	0.610	0.962	0.193	0.696	0.912
		EXT	0.306	0.553	0.976	0.246	0.611	0.961	0.193	0.697	0.912

Table S12. Leave-one-year-out temporal CV results for super learners.

$PM_{2.5}$ Speciation	Smoke Scenario	Model	Leave one year out Temporal CV								
			Daily			Monthly			Yearly		
			RMSE	R^2	Slope	RMSE	R^2	Slope	RMSE	R^2	Slope
OC	Smoke-off	XGB	0.612	0.730	1.007	0.402	0.830	1.018	0.222	0.930	1.005
		LGB	0.612	0.730	1.000	0.401	0.830	1.011	0.222	0.930	0.998
		RF	0.612	0.730	1.002	0.403	0.829	1.012	0.223	0.929	0.998
		EXT	0.609	0.733	1.010	0.401	0.830	1.018	0.223	0.930	1.004
	Smoke-on	XGB	1.384	0.559	0.964	0.776	0.705	1.006	0.454	0.829	1.050
		LGB	1.382	0.560	0.964	0.775	0.706	1.006	0.454	0.829	1.049
		RF	1.385	0.558	0.975	0.778	0.704	1.008	0.451	0.832	1.053
		EXT	1.372	0.566	0.988	0.771	0.709	1.021	0.453	0.831	1.066
EC	Smoke-off	XGB	0.191	0.752	0.978	0.124	0.848	1.012	0.080	0.920	0.975
		LGB	0.191	0.751	0.977	0.124	0.848	1.012	0.079	0.921	0.976
		RF	0.190	0.756	0.978	0.124	0.850	1.010	0.080	0.920	0.972
		EXT	0.188	0.759	0.986	0.123	0.852	1.016	0.079	0.921	0.977
	Smoke-on	XGB	0.266	0.648	0.995	0.192	0.755	1.003	0.121	0.877	0.974
		LGB	0.266	0.647	0.987	0.192	0.755	0.995	0.121	0.877	0.967
		RF	0.269	0.641	0.985	0.193	0.753	0.997	0.121	0.876	0.973
		EXT	0.266	0.647	0.993	0.192	0.755	1.000	0.120	0.878	0.972

Table S13. Model performance following the adjustment of monthly residuals in cross-validated predictions from the meta-learner using GAMs.

CV Experiments	$PM_{2.5}$ Speciation	Smoke Scenario	Model	Monthly			Yearly		
				RMSE	R^2	Slope	RMSE	R^2	Slope
**Random CV**	**OC**	Background	SL-EXT	0.338	0.880	1.008	0.135	0.974	0.992
			GAM	0.284	0.915	1.010	0.118	0.980	0.996
		Total	SL-EXT	0.373	0.904	1.035	0.150	0.971	1.013
			GAM	0.311	0.932	1.016	0.128	0.979	1.010
	**EC**	Background	SL-EXT	0.098	0.906	1.023	0.046	0.973	0.994
			GAM	0.086	0.927	1.014	0.039	0.980	0.994
		Total	SL-EXT	0.092	0.924	1.025	0.044	0.978	0.996
			GAM	0.080	0.943	1.012	0.036	0.985	0.997
**Spatial CV**	**OC**	Background	SL-EXT	0.607	0.618	1.118	0.467	0.699	1.119
			GAM	0.572	0.664	1.134	0.461	0.709	1.132
		Total	SL-EXT	0.684	0.680	1.113	0.501	0.683	1.131
			GAM	0.633	0.728	1.109	0.487	0.702	1.143
	**EC**	Background	SL-EXT	0.184	0.651	1.015	0.143	0.729	0.980
			GAM	0.175	0.685	1.022	0.139	0.745	0.991
		Total	SL-EXT	0.190	0.667	1.011	0.152	0.731	0.977
			GAM	0.180	0.700	1.017	0.147	0.748	0.992
**Temporal CV**	**OC**	Background	SL-EXT	0.401	0.830	1.018	0.223	0.930	1.004
			GAM	0.344	0.876	1.032	0.202	0.942	1.016
		Total	SL-EXT	0.494	0.831	1.052	0.252	0.918	1.028
			GAM	0.420	0.879	1.053	0.224	0.936	1.035
	**EC**	Background	SL-EXT	0.123	0.852	1.016	0.079	0.921	0.977
			GAM	0.109	0.883	1.017	0.070	0.937	0.987
		Total	SL-EXT	0.126	0.858	1.015	0.083	0.922	0.981
			GAM	0.111	0.890	1.016	0.073	0.940	0.993

Table S14. Annual deaths attributable to smoke carbonaceous $PM_{2.5}$ and related monetized damages by year and regions in the CONUS.

**Year**	**Attributable Deaths (95% Confidence Interval)**			
	**CONUS**	Northeastern	Western	Southeastern
2003	3886 (2785, 4999)	499 (221, 775)	1511 (1168, 1858)	1878 (1337, 2424)
2004	6024 (4624, 7406)	666 (459, 874)	2107 (1664, 2546)	3248 (2447, 4049)
2005	5221 (4077, 6358)	295 (167, 425)	1282 (994, 1567)	3642 (2867, 4427)
2006	5725 (4554, 6891)	451 (260, 643)	1008 (776, 1239)	4266 (3438, 5103)
2007	6954 (5668, 8239)	832 (552, 1112)	1775 (1437, 2114)	4348 (3589, 5116)
2008	9196 (7605, 10793)	1507 (969, 2043)	1922 (1560, 2282)	5767 (4839, 6714)
2009	6772 (5583, 7962)	613 (395, 833)	2062 (1687, 2436)	4099 (3383, 4818)
2010	6256 (5013, 7482)	837 (537, 1138)	1186 (933, 1439)	4232 (3466, 5009)
2011	6840 (5562, 8114)	1268 (872, 1666)	587 (446, 728)	4984 (4136, 5846)
2012	8115 (6788, 9468)	1268 (907, 1630)	779 (606, 953)	6069 (5075, 7086)
2013	7201 (5929, 8473)	1299 (914, 1687)	1428 (1171, 1683)	4472 (3708, 5239)
2014	7171 (5789, 8537)	1095 (749, 1445)	1372 (1099, 1643)	4702 (3856, 5558)
2015	5923 (4708, 7130)	869 (550, 1193)	1162 (907, 1417)	3890 (3171, 4623)
2016	8401 (6649, 10120)	2060 (1382, 2744)	1675 (1359, 1991)	4662 (3773, 5562)
2017	8554 (7024, 10082)	1546 (1086, 2009)	1392 (1084, 1702)	5618 (4700, 6549)
2018	11768 (9934, 13643)	2404 (1852, 2954)	2665 (2198, 3135)	6694 (5621, 7796)
2019	11486 (9622, 13386)	2395 (1736, 3059)	3011 (2475, 3554)	6084 (5113, 7078)
2020	8698 (7137, 10247)	1501 (1056, 1949)	1286 (1014, 1558)	5909 (4936, 6899)
Average	7455 (6058, 8852)	1189 (815, 1565)	1567 (1254, 1880)	4698 (3859, 5550)

Table S15. Annual deaths attributable to smoke carbonaceous $PM_{2.5}$ in the CONUS (results from sensitivity analyses).

Year	Attributable Deaths						
	Sensitivity Analysis 1 – Smoke Carbonaceous $PM_{2.5}$ Fractions						Sensitivity Analysis 2
	50%	60%	70%	80%	90%	95%	
2003	8186	7040	5984	5126	4441	4154	11791
2004	10532	8963	8090	7333	6651	6331	16121
2005	8761	7837	7063	6371	5762	5487	14308
2006	9235	8314	7510	6839	6244	5974	16516
2007	10657	9808	9003	8247	7565	7251	22036
2008	12765	11950	11218	10508	9830	9502	27688
2009	10026	9286	8593	7921	7311	7035	22195
2010	9979	9083	8266	7535	6856	6546	18987
2011	10423	9580	8819	8123	7464	7144	21741
2012	11171	10436	9782	9180	8628	8366	30156
2013	10629	9888	9155	8453	7802	7497	25587
2014	11071	10130	9274	8508	7800	7478	24230
2015	9798	8826	7976	7207	6527	6216	20320
2016	12649	11806	10890	9959	9120	8748	25985
2017	12402	11502	10695	9932	9215	8877	28756
2018	15117	14444	13713	13018	12371	12059	41283
2019	14970	14331	13621	12855	12143	11816	41295
2020	12516	11626	10822	10073	9371	9026	35091
Average	11160	10270	9471	8733	8061	7750	24671

Table S16. Annual monetized damages related to deaths attributable to smoke carbonaceous $PM_{2.5}$ by year and regions in the CONUS.

Year	Monetized Damages (95% Confidence Interval)				
	**VSL Estimate**: **Median** (Low, High) (Million USD/person)	**CONUS** (Billion USD)	Northeastern (Billion USD)	Western (Billion USD)	Southeastern (Billion USD)
2003	7.2 (3.4,11.0)	28.0 (13.0, 42.6)	3.6 (1.7, 5.5)	10.9 (5.1, 16.6)	13.5 (6.3, 20.6)
2004	7.4 (3.5,11.3)	44.6 (20.8, 67.9)	4.9 (2.3, 7.5)	15.6 (7.3, 23.8)	24.1 (11.2, 36.6)
2005	7.5 (3.5,11.5)	39.4 (18.4, 60.0)	2.2 (1.0, 3.4)	9.7 (4.5, 14.7)	27.5 (12.8, 41.8)
2006	7.8 (3.6,11.9)	44.6 (20.8, 67.9)	3.5 (1.6, 5.3)	7.9 (3.7, 12.0)	33.2 (15.5, 50.6)
2007	8.1 (3.8,12.3)	56.0 (26.2, 85.3)	6.7 (3.1, 10.2)	14.3 (6.7, 21.8)	35.0 (16.4, 53.3)
2008	8.4 (3.9,12.7)	77.0 (35.9, 117.1)	12.6 (5.9, 19.2)	16.1 (7.5, 24.5)	48.3 (22.5, 73.5)
2009	8.6 (4.0 ,13.1)	58.2 (27.1, 88.5)	5.3 (2.5, 8.0)	17.7 (8.3, 27.0)	35.2 (16.4, 53.6)
2010	8.7 (4.0 ,13.2)	54.1 (25.3, 82.4)	7.2 (3.4, 11.0)	10.3 (4.8, 15.6)	36.6 (17.1, 55.7)
2011	8.8 (4.1,13.3)	60.0 (28.0, 91.3)	11.1 (5.2, 16.9)	5.1 (2.4, 7.8)	43.7 (20.4, 66.5)
2012	8.9 (4.2,13.6)	72.4 (33.8, 110.2)	11.3 (5.3, 17.2)	7.0 (3.2, 10.6)	54.2 (25.3, 82.4)
2013	9.0 (4.2,13.7)	64.8 (30.2, 98.7)	11.7 (5.5, 17.8)	12.8 (6.0, 19.6)	40.3 (18.8, 61.3)
2014	9.2 (4.3,14.0)	65.8 (30.7, 100.1)	10.0 (4.7, 15.3)	12.6 (5.9, 19.2)	43.1 (20.1, 65.7)
2015	9.4 (4.4,14.3)	55.5 (25.9, 84.5)	8.1 (3.8, 12.4)	10.9 (5.1, 16.6)	36.5 (17.0, 55.5)
2016	9.7 (4.5,14.7)	81.2 (37.9, 123.6)	19.9 (9.3, 30.3)	16.2 (7.6, 24.6)	45.0 (21.0, 68.6)
2017	10.0 (4.7,15.2)	85.4 (39.8, 130.0)	15.4 (7.2, 23.5)	13.9 (6.5, 21.1)	56.1 (26.2, 85.4)
2018	10.3 (4.8,15.7)	121.0 (56.5, 184.2)	24.7 (11.5, 37.6)	27.4 (12.8, 41.7)	68.8 (32.1, 104.8)
2019	10.6 (5.0 ,16.2)	122.3 (57.1, 186.2)	25.5 (11.9, 38.8)	32.1 (15.0, 48.8)	64.8 (30.2, 98.6)
2020	11.4 (5.3,17.4)	99.2 (46.3, 151.1)	17.1 (8.0, 26.1)	14.7 (6.8, 22.3)	67.4 (31.5, 102.6)
Average	/	68.3 (31.9, 104.0)	11.2 (5.2, 17.0)	14.2 (6.6, 21.6)	43.0 (20.0, 65.4)

Note: the VSL estimates were adjusted for each specific dollar year. The median VSL is used for calculating the monetized damages and 95% confidence intervals.

Table S17. Annual deaths attributable to smoke carbonaceous $PM_{2.5}$ and related monetized damages by year in SC.

Year	Attributable Deaths	VSL Estimate: **Median** (Low, High) (Million CAD/person)	Monetized Damages (Billion CAD)
2003	163 (85, 241)	6.0 (3.2,8.8)	1.0 (0.5, 1.4)
2004	211 (110, 311)	6.1 (3.3,8.9)	1.3 (0.7, 1.9)
2005	122 (64, 180)	6.2 (3.4,9.1)	0.8 (0.4, 1.1)
2006	135 (71, 199)	6.4 (3.4,9.3)	0.9 (0.4, 1.3)
2007	198 (104, 292)	6.5 (3.5,9.5)	1.3 (0.7, 1.9)
2008	234 (122, 346)	6.7 (3.6,9.7)	1.6 (0.8, 2.3)
2009	147 (77, 217)	6.7 (3.6,9.8)	1.0 (0.5, 1.4)
2010	175 (92, 258)	6.8 (3.7,9.9)	1.2 (0.6, 1.8)
2011	240 (125, 354)	7.0 (3.8,10.2)	1.7 (0.9, 2.5)
2012	210 (110, 311)	7.1 (3.8,10.4)	1.5 (0.8, 2.2)
2013	270 (141, 399)	7.2 (3.9,10.5)	1.9 (1.0, 2.9)
2014	273 (143, 403)	7.3 (3.9,10.7)	2.0 (1.0, 2.9)
2015	198 (104, 293)	7.4 (4.0,10.8)	1.5 (0.8, 2.2)
2016	397 (208, 586)	7.5 (4.0,11.0)	3.0 (1.6, 4.4)
2017	208 (109, 308)	7.6 (4.1,11.1)	1.6 (0.8, 2.3)
2018	602 (315, 888)	7.8 (4.2,11.4)	4.7 (2.5, 6.9)
2019	578 (303, 853)	7.9 (4.3,11.6)	4.6 (2.4, 6.8)
2020	309 (162, 456)	8.0 (4.3,11.7)	2.5 (1.3, 3.6)
Average	259 (136, 383)	/	1.9 (1.0, 2.8)

Note: the VSL estimates were adjusted for each specific dollar year. The median VSL is used for calculating the monetized damages and 95% confidence intervals.

Supplemental Figures

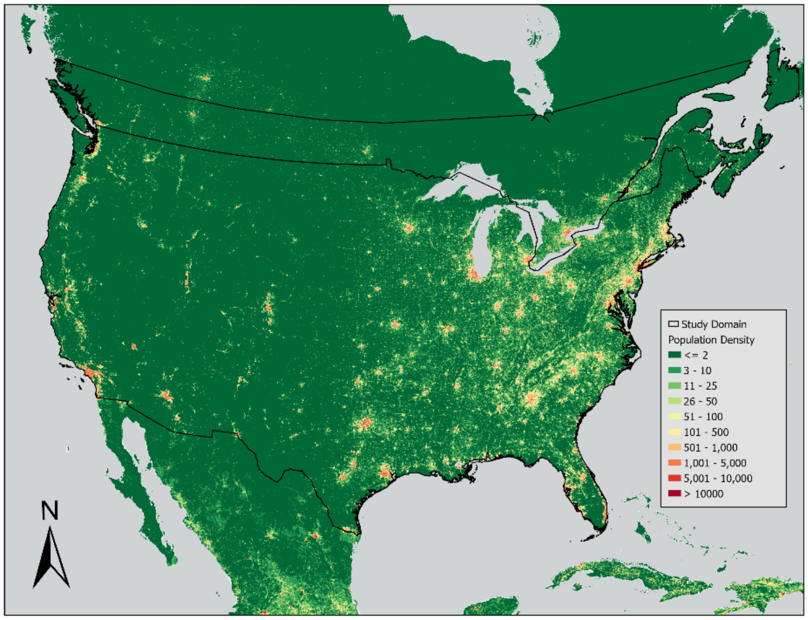

Fig. S1. Landscan population density in 2019.

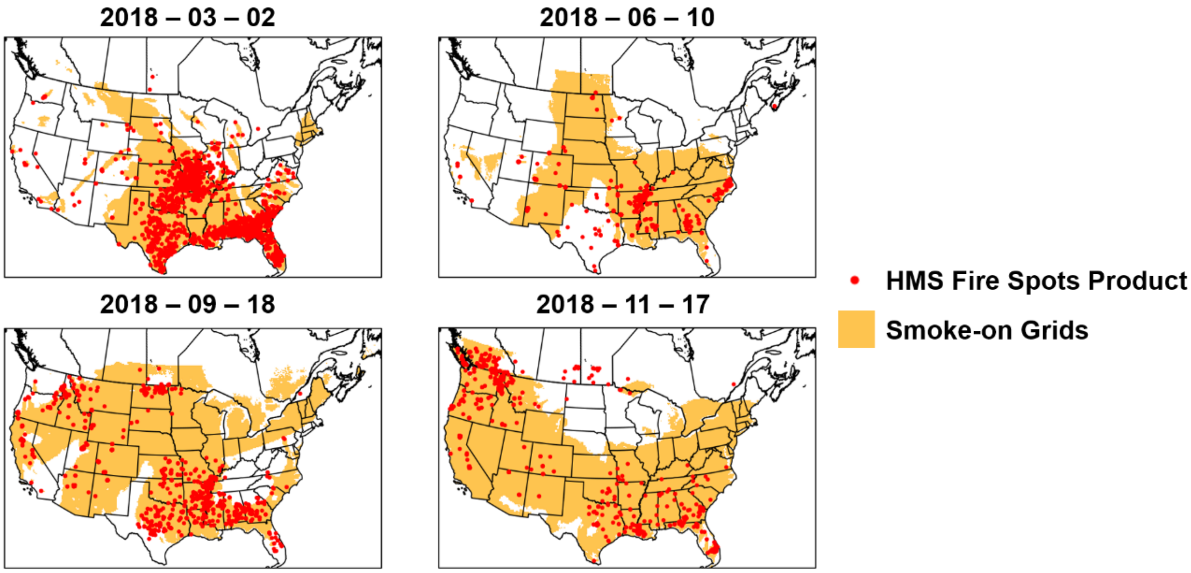

Fig. S2. Maps of Smoke-on grids and HMS fire spots products for four selected days in 2018.

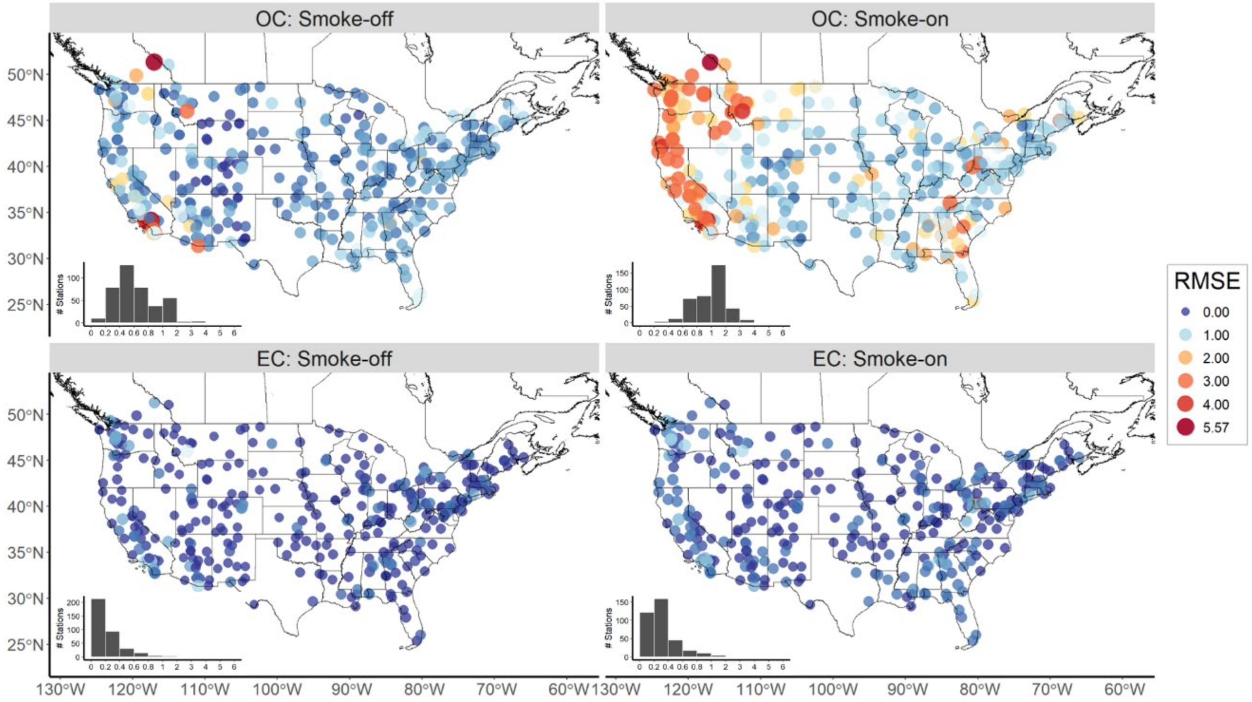

Fig. S3. Maps of 10-fold clustered spatial CV RMSE at monitoring stations for $PM_{2.5}$ OC and EC.

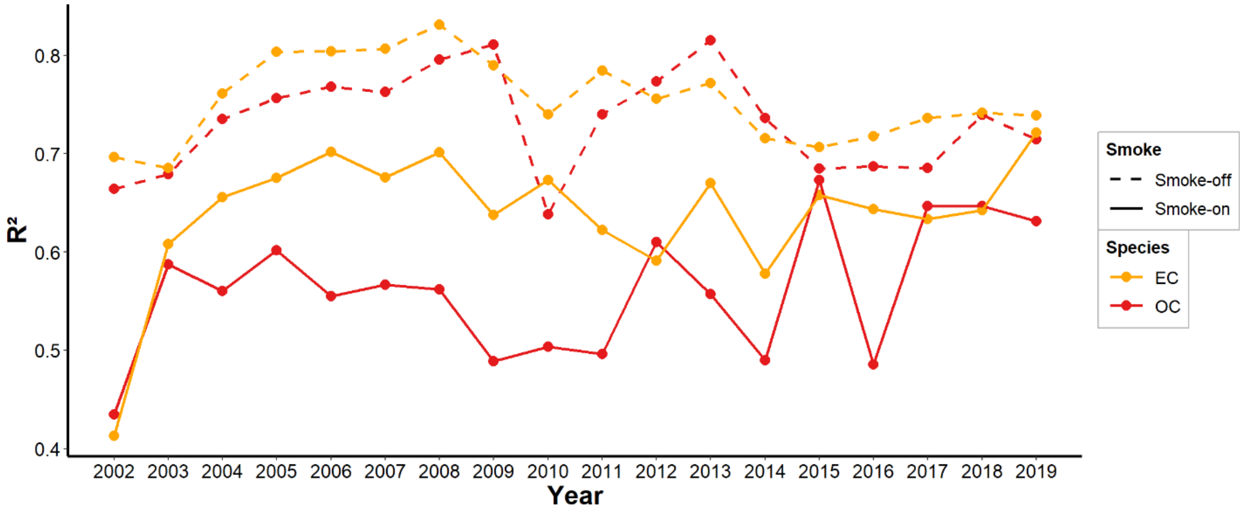

Fig. S4. Leave-one-year-out temporal CV R^2 from 2002 to 2019.

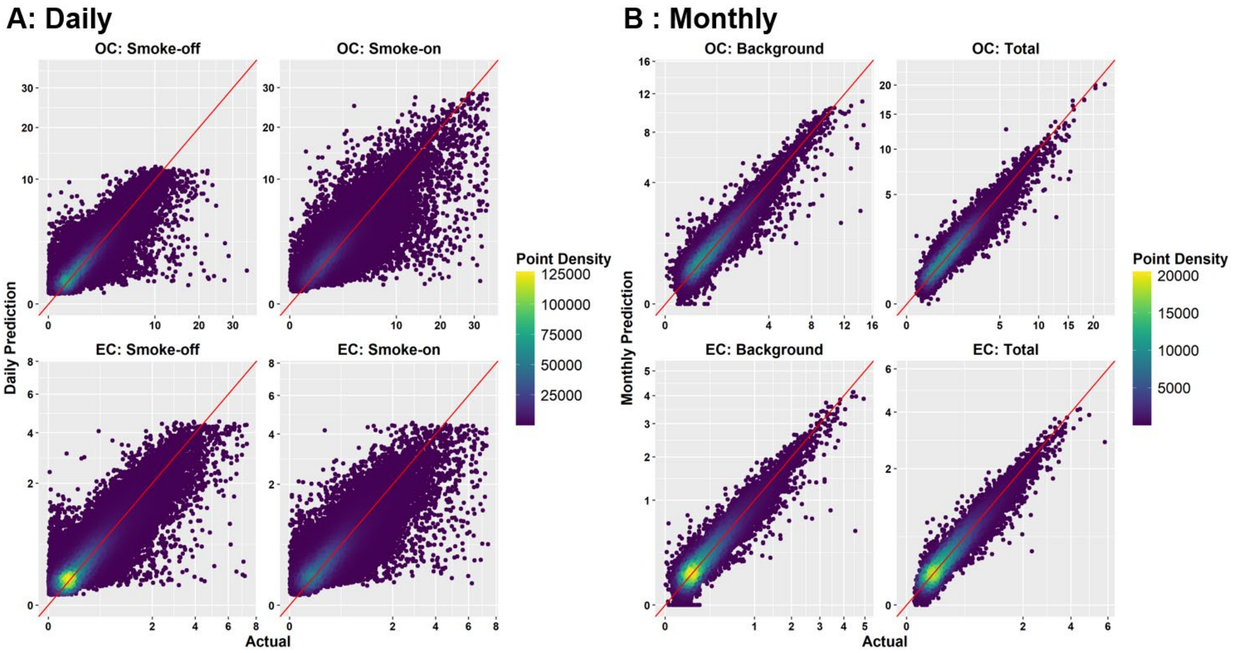

Fig. S5. Scatter plots of 10-fold random CV predictions (Unit: μg/m^3). **A.** Daily predictions from EXT meta-learners. **B.** Monthly predictions from GAMs. **Note**: Red lines indicate the 1:1 line.

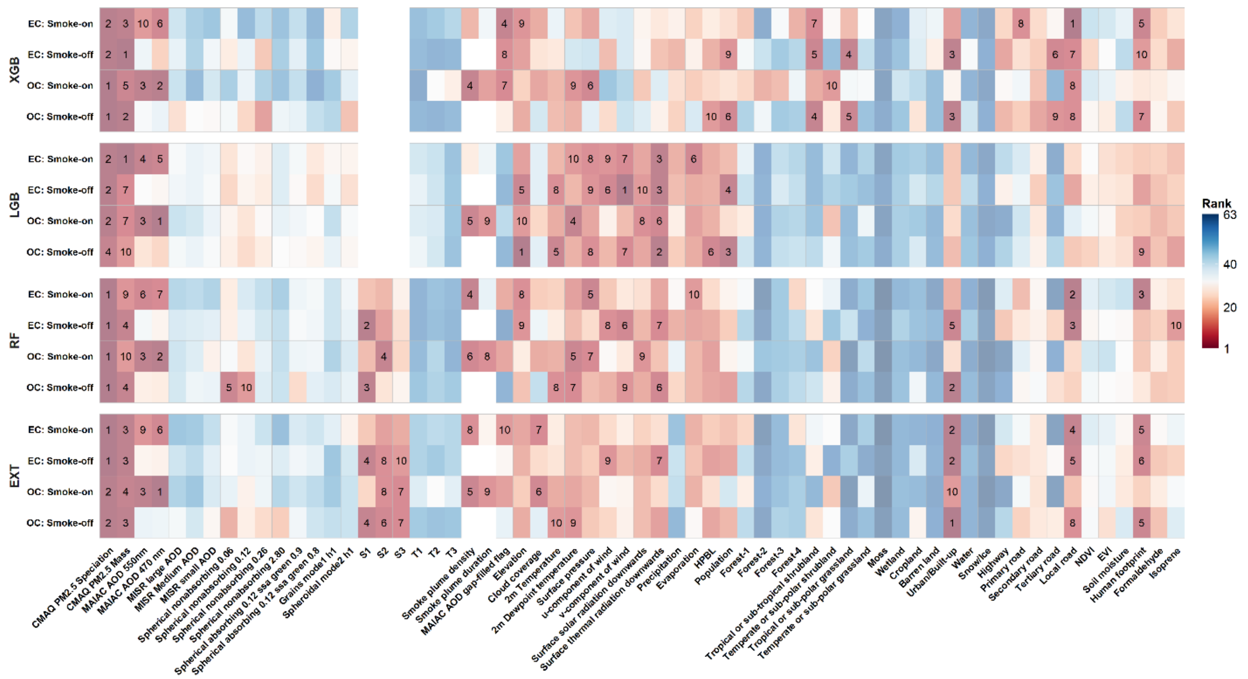

Fig. S6. Feature importance rankings across four base learners for carbonaceous $PM_{2.5}$ (OC and EC) under two smoke scenarios. The top 10 predictors for each model are labeled. Note: the three spherical coordinates [S1, S2, S3] were excluded from XGB and LGB models to prevent overfitting. Feature importance is ranked using impurity-based (for RF and EXT) and gain-based (for XGB and LGB) methods.

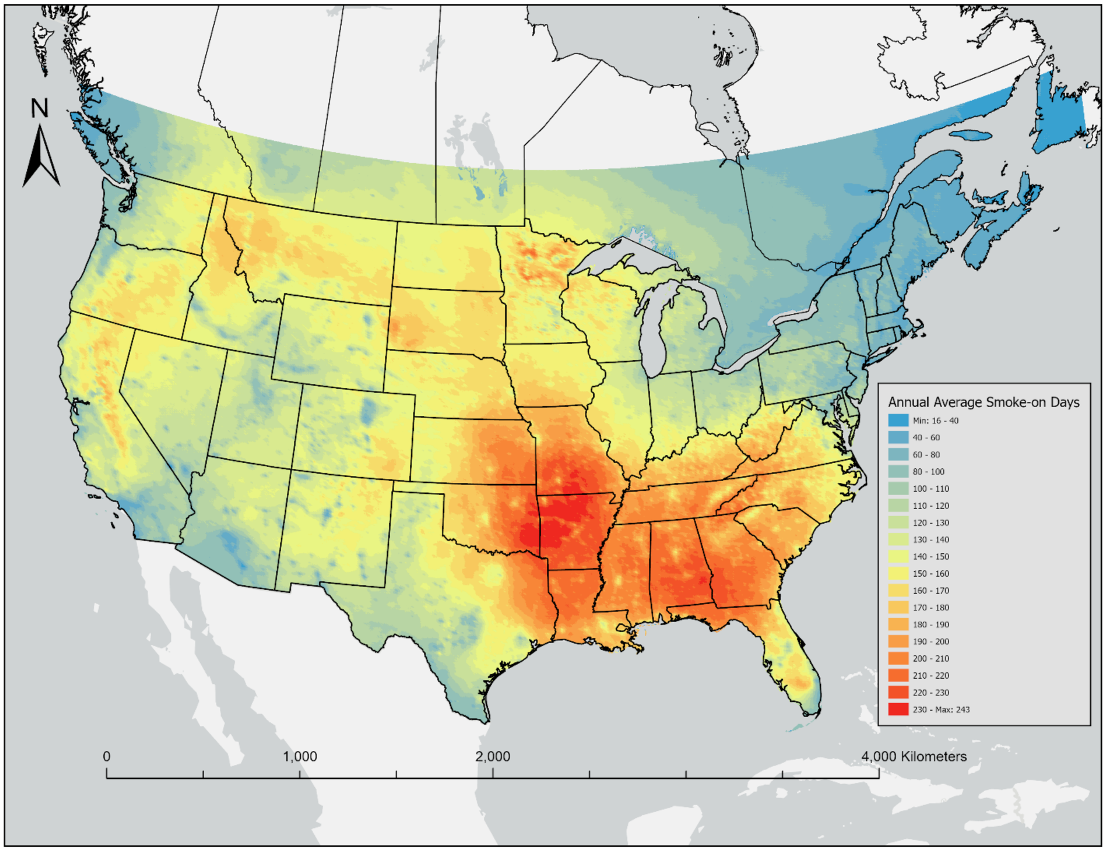

Fig. S7. Long-term average annual Smoke-on days across the study domain.

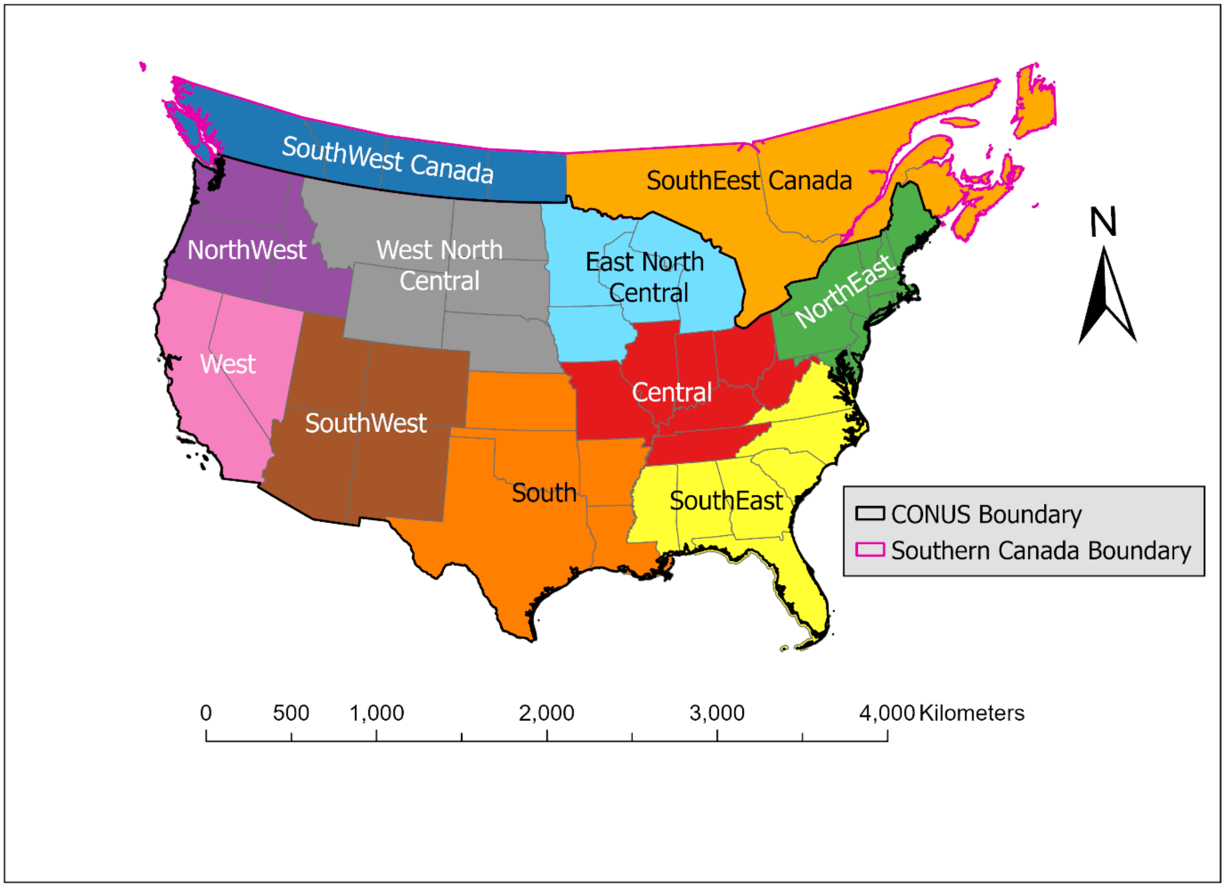

Fig. S8. Climate region classification based on climate types obtained from the NOAA and Canada's Changing Climate Report.[23, 24]

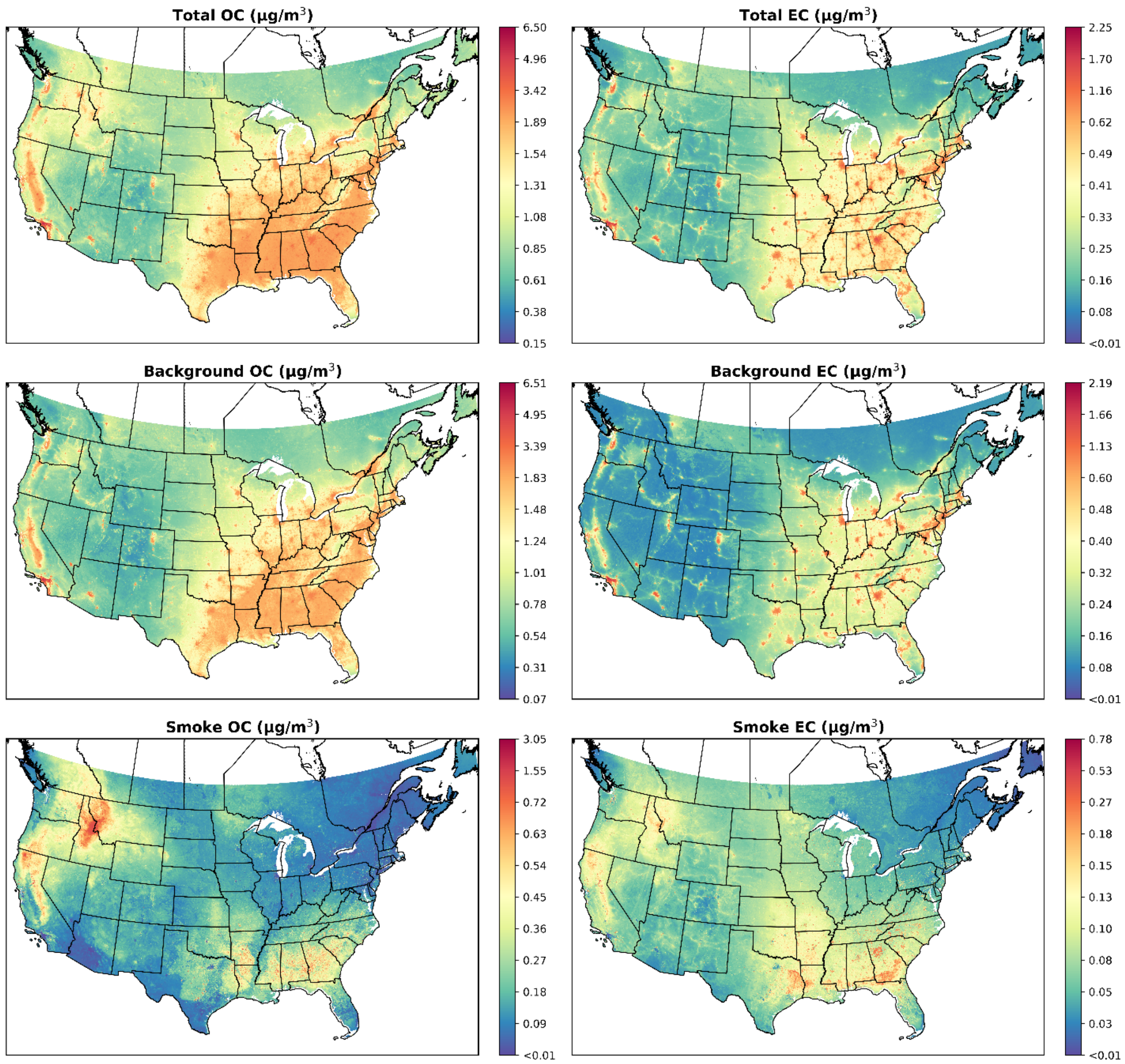

Fig. S9. Long-term average annual concentration of background, total, and smoke $PM_{2.5}$ OC and EC from 2002 to 2019 (Unit: μg/m^3).

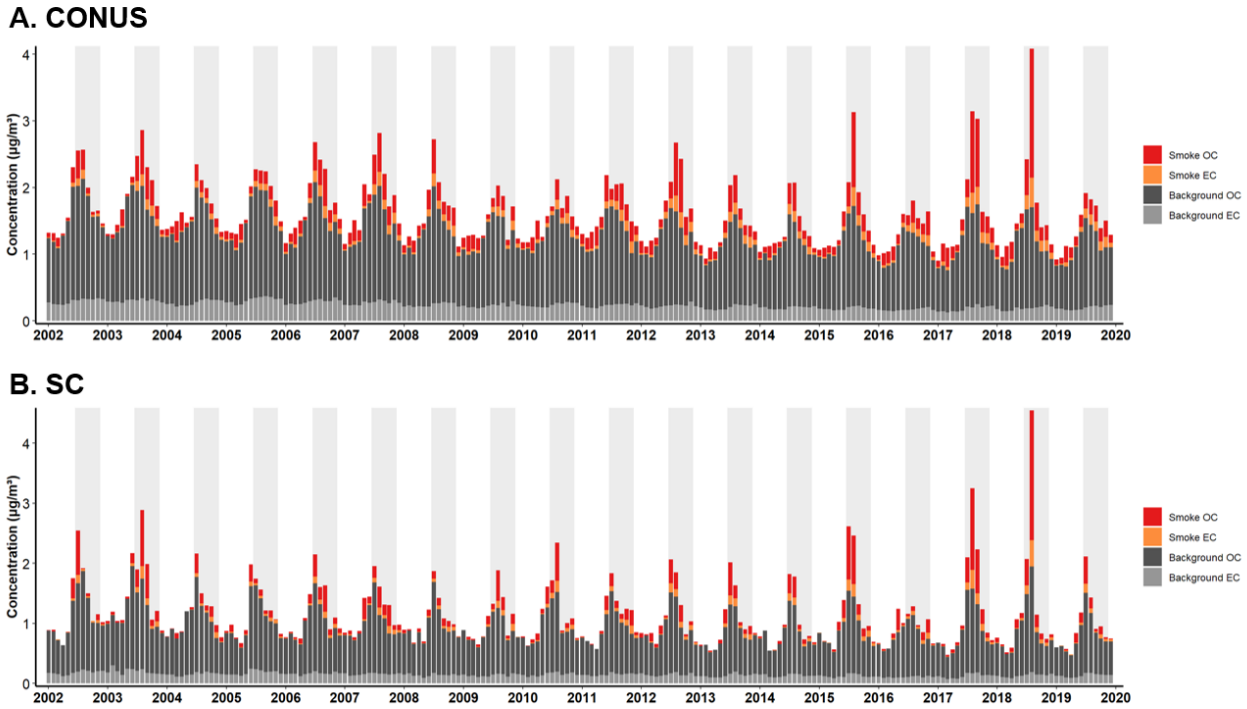

Fig. S10. Monthly mean concentrations of background and smoke carbonaceous $PM_{2.5}$ in the CONUS (subplot-**A**) and SC (subplot-**B**) (Unit: μg/m^3). **Note**: The grey background indicates the peak months of wildfire season from July to November.

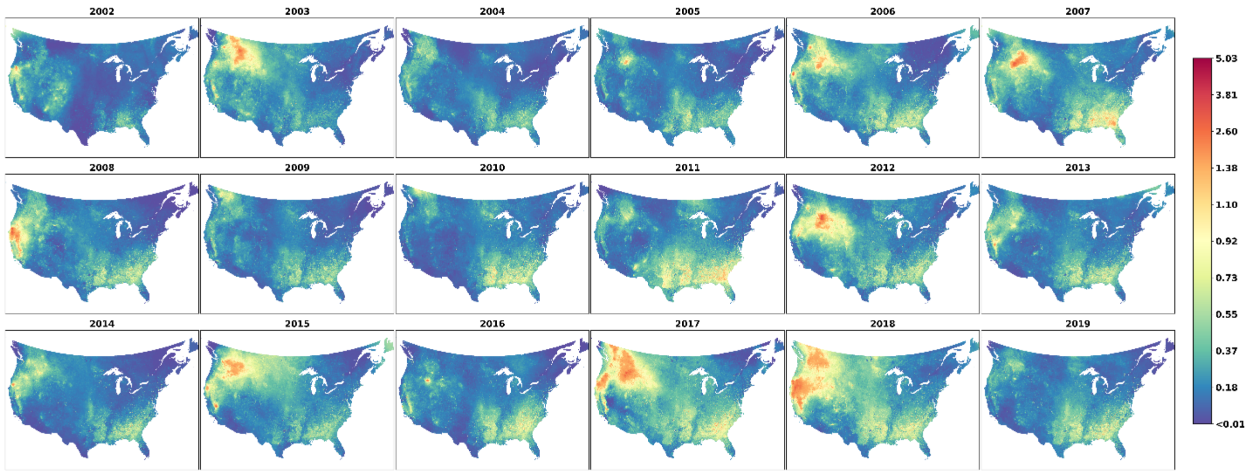

Fig. S11. Annual smoke-specific carbonaceous $PM_{2.5}$ from 2002 to 2019 across the study domain.

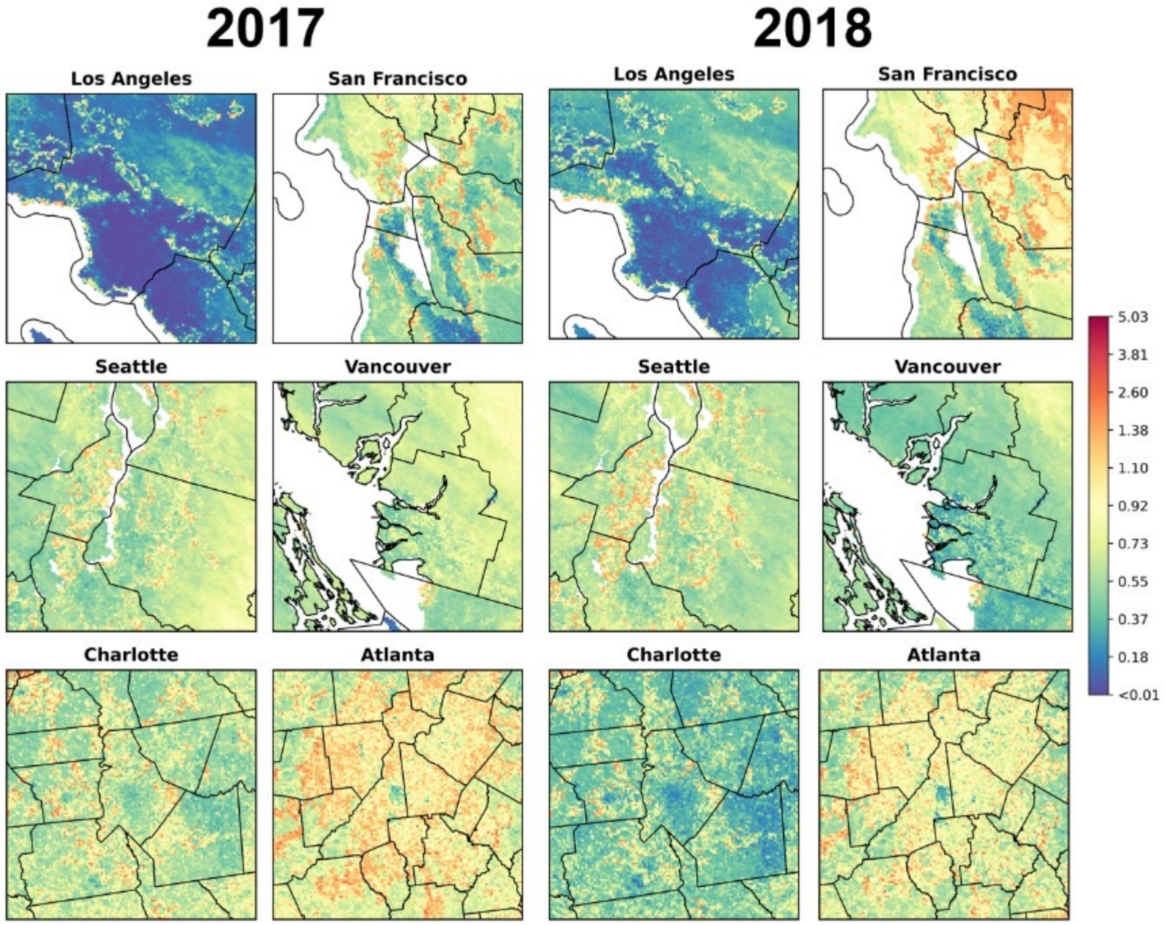

Fig. S12. Annual smoke-specific carbonaceous $PM_{2.5}$ in six major urban centers during 2017 and 2018.

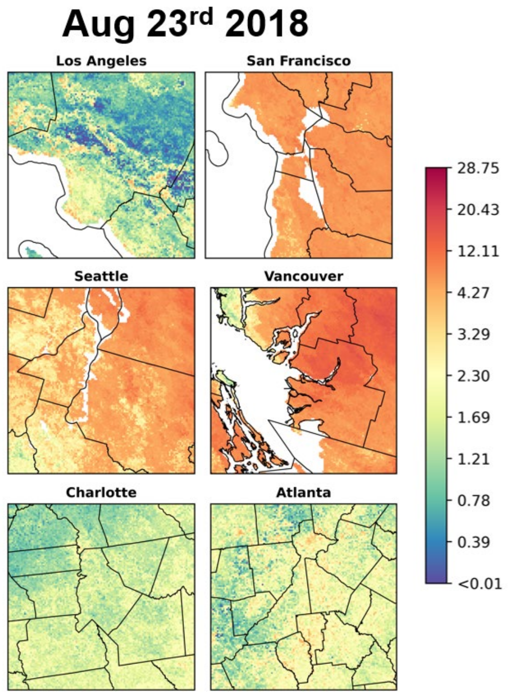

Fig. S13. Smoke-specific carbonaceous $PM_{2.5}$ in six major urban centers on August 23rd 2018.

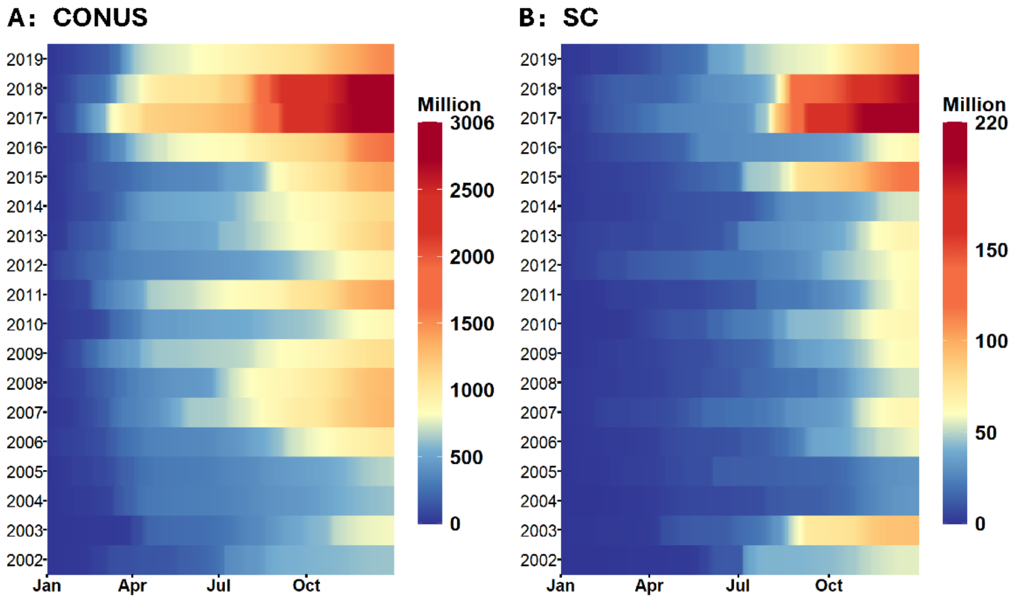

Fig. S14. Cumulative person-days affected by heavy fire smoke by year in the CONUS (subplot-**A**) and SC (subplot-**B**) (Unit: million person-day).

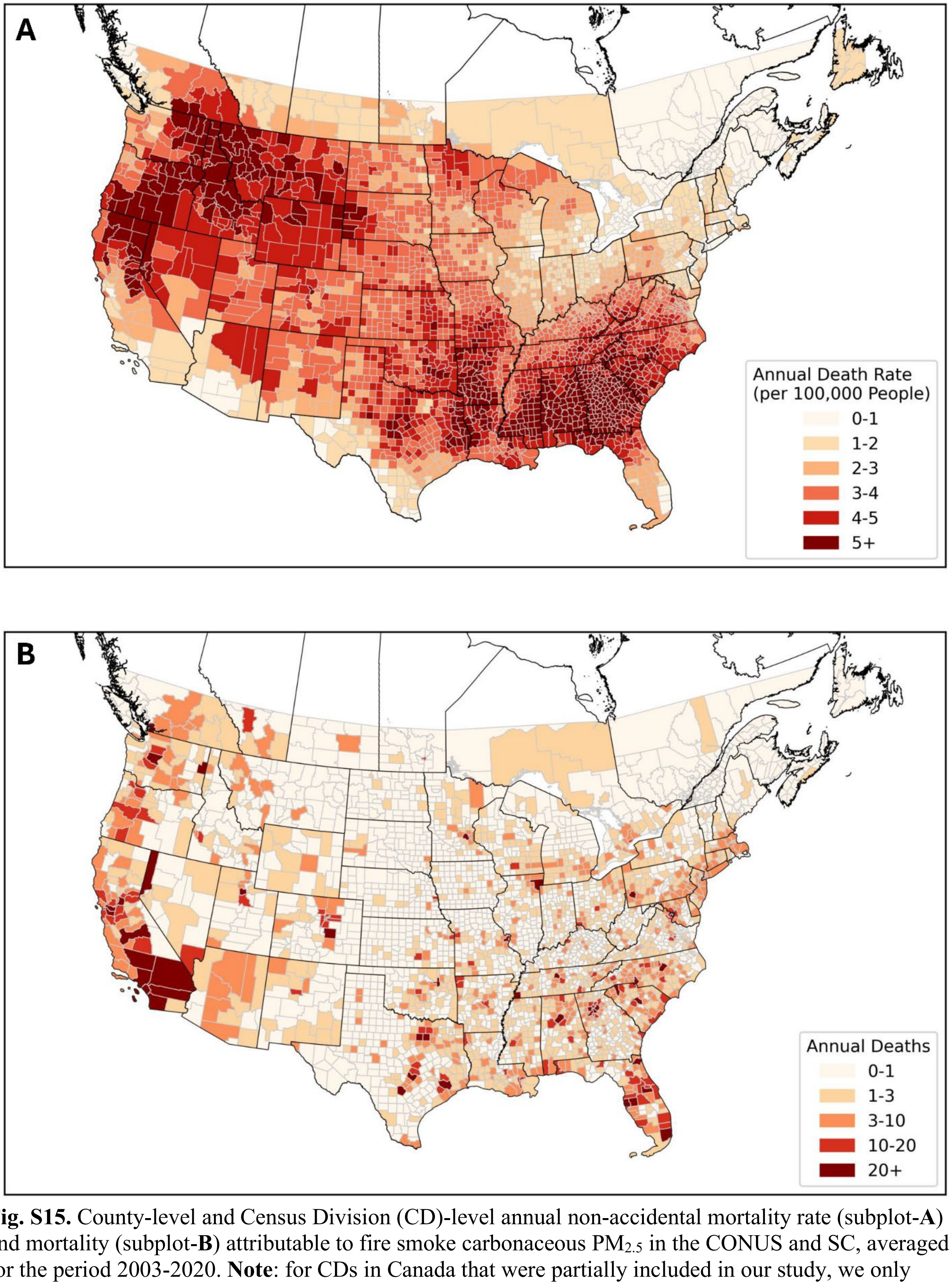

Fig. S15. County-level and Census Division (CD)-level annual non-accidental mortality rate (subplot-**A**) and mortality (subplot-**B**) attributable to fire smoke carbonaceous $PM_{2.5}$ in the CONUS and SC, averaged for the period 2003-2020. **Note**: for CDs in Canada that were partially included in our study, we only consider the population within the study domain.

Supplemental References

(1) Mu, H.; Li, X.; Wen, Y.; Huang, J.; Du, P.; Su, W.; Miao, S.; Geng, M. A global record of annual terrestrial Human Footprint dataset from 2000 to 2018. *Scientific Data* **2022**, *9* (1), 176.
(2) Wei, J.; Li, Z.; Chen, X.; Li, C.; Sun, Y.; Wang, J.; Lyapustin, A.; Brasseur, G. P.; Jiang, M.; Sun, L.; Wang, T.; Jung, C. H.; Qiu, B.; Fang, C.; Liu, X.; Hao, J.; Wang, Y.; Zhan, M.; Song, X.; Liu, Y. Separating Daily 1 km $PM_{2.5}$ Inorganic Chemical Composition in China since 2000 via Deep Learning Integrating Ground, Satellite, and Model Data. *Environmental Science & Technology* **2023**, *57* (46), 18282-18295. DOI: 10.1021/acs.est.3c00272.
(3) Chawla, N. V.; Bowyer, K. W.; Hall, L. O.; Kegelmeyer, W. P. SMOTE: synthetic minority over-sampling technique. *Journal of artificial intelligence research* **2002**, *16*, 321-357.
(4) Chen, T.; Guestrin, C. Xgboost: A scalable tree boosting system. In *Proceedings of the 22nd acm sigkdd international conference on knowledge discovery and data mining*, 2016; pp 785-794.
(5) Ke, G.; Meng, Q.; Finley, T.; Wang, T.; Chen, W.; Ma, W.; Ye, Q.; Liu, T.-Y. Lightgbm: A highly efficient gradient boosting decision tree. *Advances in neural information processing systems* **2017**, *30*.
(6) Breiman, L. Random forests. *Machine learning* **2001**, *45*, 5-32.
(7) Geurts, P.; Ernst, D.; Wehenkel, L. Extremely randomized trees. *Machine learning* **2006**, *63*, 3-42.
(8) Zamani Joharestani, M.; Cao, C.; Ni, X.; Bashir, B.; Talebiesfandarani, S. $PM_{2.5}$ prediction based on random forest, XGBoost, and deep learning using multisource remote sensing data. *Atmosphere* **2019**, *10* (7), 373.
(9) Zhang, X.; Jiang, X.; Li, Y. Prediction of air quality index based on the SSA-BiLSTM-LightGBM model. *Scientific Reports* **2023**, *13* (1), 5550.
(10) Eslami, E.; Salman, A. K.; Choi, Y.; Sayeed, A.; Lops, Y. A data ensemble approach for real-time air quality forecasting using extremely randomized trees and deep neural networks. *Neural Computing and Applications* **2020**, *32*, 7563-7579.
(11) Hastie, T. J. Generalized additive models. *Statistical models in S* **2017**, 249-307.
(12) Solomon, P. A.; Crumpler, D.; Flanagan, J. B.; Jayanty, R.; Rickman, E. E.; McDade, C. E. US national $PM_{2.5}$ chemical speciation monitoring networks—CSN and IMPROVE: description of networks. *Journal of the Air & Waste Management Association* **2014**, *64* (12), 1410-1438.
(13) Dabek-Zlotorzynska, E.; Dann, T. F.; Martinelango, P. K.; Celo, V.; Brook, J. R.; Mathieu, D.; Ding, L.; Austin, C. C. Canadian National Air Pollution Surveillance (NAPS) $PM_{2.5}$ speciation program: Methodology and $PM_{2.5}$ chemical composition for the years 2003–2008. *Atmospheric Environment* **2011**, *45* (3), 673-686.
(14) Centers for Disease Control and Prevention; National Center for Health Statistics; National Vital Statistics System. Mortality 1999-2020 on CDC WONDER Online Database. 2021.
(15) Turpin, B. J.; Lim, H.-J. Species contributions to $PM_{2.5}$ mass concentrations: Revisiting common assumptions for estimating organic mass. *Aerosol Science & Technology* **2001**, *35* (1), 602-610.
(16) Simon, H.; Bhave, P.; Swall, J.; Frank, N.; Malm, W. Determining the spatial and seasonal variability in OM/OC ratios across the US using multiple regression. *Atmospheric Chemistry and Physics* **2011**, *11* (6), 2933-2949.
(17) Appel, K. W.; Bash, J. O.; Fahey, K. M.; Foley, K. M.; Gilliam, R. C.; Hogrefe, C.; Hutzell, W. T.; Kang, D.; Mathur, R.; Murphy, B. N.; Napelenok, S. L.; Nolte, C. G.; Pleim, J. E.; Pouliot, G. A.; Pye, H. O. T.; Ran, L.; Roselle, S. J.; Sarwar, G.; Schwede, D. B.; Sidi, F. I.; Spero, T. L.; Wong, D. C. The Community Multiscale Air Quality (CMAQ) model versions 5.3 and 5.3.1: system updates and evaluation. *Geosci. Model Dev.* **2021**, *14* (5), 2867-2897. DOI: 10.5194/gmd-14-2867-2021.
(18) Pye, H. O.; Luecken, D. J.; Xu, L.; Boyd, C. M.; Ng, N. L.; Baker, K. R.; Ayres, B. R.; Bash, J. O.; Baumann, K.; Carter, W. P. Modeling the current and future roles of particulate organic nitrates in the southeastern United States. *Environmental Science & Technology* **2015**, *49* (24), 14195-14203.
(19) Xu, L.; Pye, H. O.; He, J.; Chen, Y.; Murphy, B. N.; Ng, N. L. Experimental and model estimates of the contributions from biogenic monoterpenes and sesquiterpenes to secondary organic aerosol in the southeastern United States. *Atmospheric chemistry and physics* **2018**, *18* (17), 12613-12637.

(20) Pye, H. O.; Murphy, B. N.; Xu, L.; Ng, N. L.; Carlton, A. G.; Guo, H.; Weber, R.; Vasilakos, P.; Appel, K. W.; Budisulistiorini, S. H. On the implications of aerosol liquid water and phase separation for organic aerosol mass. *Atmospheric Chemistry and Physics* **2017**, *17* (1), 343-369.
(21) Sarwar, G.; Gantt, B.; Schwede, D.; Foley, K.; Mathur, R.; Saiz-Lopez, A. Impact of enhanced ozone deposition and halogen chemistry on tropospheric ozone over the Northern Hemisphere. *Environmental science & technology* **2015**, *49* (15), 9203-9211.
(22) Ma, Y.; Zang, E.; Liu, Y.; Wei, J.; Lu, Y.; Krumholz, H. M.; Bell, M. L.; Chen, K. Long-term exposure to wildland fire smoke $PM_{2.5}$ and mortality in the contiguous United States. *Proceedings of the National Academy of Sciences* **2024**, *121* (40), e2403960121. DOI: doi:10.1073/pnas.2403960121.
(23) Karl, T.; Koss, W. J. Regional and national monthly, seasonal, and annual temperature weighted by area, 1895-1983. **1984**, Miscellaneous.
(24) Bush, E.; Lemmen, D. S. *Canada's Changing Climate Report*. Government of Canada, 2019. https://changingclimate.ca/CCCR2019/ (accessed.
